# Highly Efficient Photoinitiation Systems Based on Dibenzo[a,c]phenazine Sensitivity to Visible Light for Dentistry

**DOI:** 10.3390/ma17112597

**Published:** 2024-05-28

**Authors:** Ilona Pyszka, Beata Jędrzejewska

**Affiliations:** Faculty of Chemical Technology and Engineering, Bydgoszcz University of Science and Technology, ul. Seminaryjna 3, 85-326 Bydgoszcz, Poland

**Keywords:** dibenzo[a,c]phenazine derivatives, dye photoinitiators, photopolymerization, dental filling compositions

## Abstract

In this work, photoinitiation systems based on dibenzo[a,c]phenazine sensitivity to visible light were designed for their potential application in dentistry. Modification of the structure of dibenzo[a,c]phenazine consisted of introducing electron-donating and electron-withdrawing substituents and heavy atoms into position 11. The synthesized compounds are able to absorb radiation emitted by dental lamps during photoinitiation of the polymerization process. In the presence of acrylates, dibenzo[a,c]phenazines show excellent photoinitiating abilities in systems containing an electron donor or a hydrogen-atom donor as a second component. The developed systems initiate the polymerization process comparable to a commercial photoinitiator, i.e., camphorquinone. Moreover, the performed studies showed a significant shortening of the polymerization time and a reduction in the amount of light absorber. This indicates that polymeric materials are obtained at a similar rate despite a significant reduction in the concentration of the newly developed two-component photoinitiating systems.

## 1. Introduction

Over the last decades, polymer photochemistry has become a field of central importance in polymer science and technology. Applications such as photopolymerization, photocrosslinking, photostabilization, and solar energy devices have evolved from basic research to industrial products [1]. Photopolymerization is the process of linking monomers into polymers under the influence of a photoinitiator, which, under UV–visible irradiation, decomposes to form active centers that initiate the polymerization reaction. Currently, photochemically initiated processes constitute the basis for most modern technologies for the production of polymeric materials [2,3]. Moreover, the synthesis of polymeric materials carried out by photopolymerization is one of the most productive methods. That is why this technique is widely used and constantly modernized [4,5,6,7,8]. Nowadays, its most popular applications are medicine, including dentistry, coating, and 3D/4D printing [8,9,10,11,12,13,14,15,16,17,18,19,20,21,22]. Due to low energy consumption, a lack of solvents, and a high rate at ambient temperatures, photopolymerization is considered an ecological method compared to other techniques.

The two most important methods of photochemically initiated chain reactions are widely used in industry, i.e., radical and cationic polymerization [23]. They differ primarily in the mechanism but also in the type of monomers and initiators used.

Radical polymerization takes place in the presence of acrylate and methacrylate monomers [24], which are relatively highly reactive and can produce materials with desired properties. Photoinitiators for this type of polymerization must be sensitive to light in the UV–visible region [2,25]. Following the absorption of a quantum of electromagnetic energy, radicals are generated from the initiator, which can start the polymerization process [26]. Depending on the mechanism of radical formation, there are two main groups of photoinitiators, i.e., type I and type II [27,28,29]. In type I photoinitiators, upon irradiation, a direct bond homolytic cleavage occurs resulting in the formation of two highly reactive radicals [30,31]. Generally, these photoinitiators, on the one hand, should have a bond with a dissociation energy lower than the excitation energy of the reactive excited state, and, on the other hand, high enough to ensure thermal stability [1,32]. Depending on the type and position of the functional group, photodissociation takes place in the α (α-cleavage photoinitiators) or β (β-cleavage photoinitiators) position in relation to the carbonyl group. There are also known type I photoinitiators containing weak bonds between heteroatoms, e.g., O–O, S–S, N–S, C–N, and others [30,31]. Type II photoinitiators generate radicals in the electron transfer process (PET) between the photoinitiator and co-initiator [33,34,35,36,37], or in the hydrogen-atom transfer process [37,38,39]. Type II photoinitiators include aromatic ketones, such as benzophenone [40], thioxanthone [41], ketocoumarin [42], and their derivatives, as well as α-diketones (e.g., camphorquinone) [43,44,45] and various dyes: cyanines [46], phenazines [47], quinoxalines [37], and many others [36,48,49,50,51,52]. Co-initiators are compounds that react with an excited initiator, resulting in the formation of a radical capable of initiating free-radical polymerization. These are most often aromatic amines or sulfur compounds [25,43,53]. 

Photopolymerization is now widely used in dentistry for in vivo hardening of polymer fillings that recreate lost hard-tooth tissues [54,55]. After introducing the photosensitive monomer composition into the defect, the material layers are hardened using light. Polymer composite materials used for fillings consist of a mixture of single- and multifunctional monomers, initiator–co-initiator systems (camphorquinone–aromatic amine), and reinforcing fillers (quartz, silicate glass, silicon nitrides, etc.). In addition to fillers, other additives such as antioxidants, photostabilizers, and dyes are sometimes added. They can significantly influence the course of polymerization because most of them absorb UV radiation or visible light. Radiation may also be dispersed and limited in its penetration into the polymerization mixture, which is why cross-linking photopolymerization is usually conducted in thin layers from 0.1 µm to 1–5 mm.

Modern dentistry is looking for universal, high-performance photoinitiating systems useful for obtaining composite materials that rebuild hard tooth tissues. The desired properties of composites can be obtained by modifying the composition of the polymer matrix. Therefore, attempts have been made to develop new, highly efficient photoinitiating systems that will demonstrate better light sensitivity to light sources used in dentistry. To achieve this goal, compounds based on a dibenzo[a,c]phenazine skeleton were synthesized, the structures of which were modified to improve absorption properties in the visible region. Additionally, currently used aromatic amines acting as co-initiators were replaced with acetic acid derivatives. This significantly accelerated the photopolymerization process and shortened its duration, as well as reduced the amount of photoinitiator used.

## 2. Materials and Methods

### 2.1. Reagents

All reagents and solvents, namely 1,2-diaminobenzene, 9,10-phenanthrenequinone, 4-bromo-1,2-diaminobenzene, 4-iodo-2-nitroaniline, 4-methyl-*o*-phenylenediamine, 3,4-diaminobenzoic acid, 3,4-diaminobenzonitrile, 4-methoxy-*o*-phenylenediamine dihydrochloride, methyl 3,4-diaminobenzoate, 3,4-diaminobenzophenone, sodium hydroxide, acetic acid, ethyl acetate, cyclohexane, 1-methyl-2-pyrrolidinone (MP), chloroform, deuterated chloroform (CDCl_3_), acetonitrile (CD_3_CN), and dimethyl sulfoxide (DMSO-*d*_6_) were provided by Alfa Aesar Co. (Haverhill, MA, USA) or Merck Co. (Rahway, NJ, USA).

A commercial photoinitiator, camphorquinone (CQ), the co-initiators (phenylthio)acetic acid (PhTAA), phenylacetic acid (PhAA), *N*,*N*-dimethylaniline (DMA), and 2-mercaptobenzoxazole (MBO), and the monomer trimethylolpropane triacrylate (TMPTA) were purchased from Merck Co.

**Figure 1:**
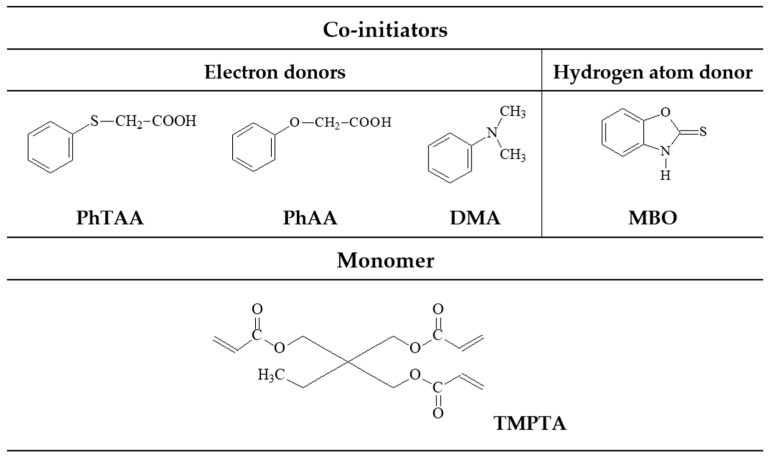
Co-initiators and monomers used in the research.

Figure 1 illustrates the chemical structures of the co-initiators and monomer used in the study. 

### 2.2. Synthesis

**Figure 2:**
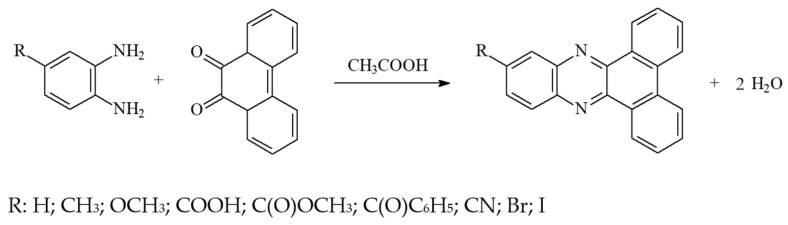
Synthesis of dibenzo[a,c]phenazine (DBPh1–DBPh9) derivatives.

Many research groups have attempted to synthesize dibenzo[a,c]phenazine and its derivatives using various reagents [4,56,57,58,59,60]. Our proposed method for obtaining dibenzo[a,c]phenazine derivatives is simple and easy to perform. Moreover, the efficiency of about 80–93% is high. The synthesis proceeded according to the reaction shown in Figure 2.

To assess the progress of the synthesis, thin-layer chromatography was performed on silica gel 60F 254 using chloroform as the eluent. The products were identified spectroscopically. The ^1^H and ^13^C NMR spectra are shown as images before Figure S1 in the ESI file whereas their interpretation is presented below. 

The new peak at 140.0–160.0 ppm in the ^13^C NMR spectra indicating the formation of the imine group (CN) containing a carbon–nitrogen double bond, as well as the disappearance of the signal at 3.0–5.0 ppm in the ^1^H NMR spectra originating from the amino group confirms that the intended compounds have been obtained.

#### 2.2.1. Procedure for Obtaining Dibenzo[a,c]phenazine (DBPh1) and Its Derivatives (DBPh2–DBPh9)

##### DBPh1: Dibenzo[a,c]phenazine

Dibenzo[a,c]phenazine (DBPh1) was prepared by refluxing 0.519 g (4.8 mmol) 1,2-diaminobenzene with 1.0 g (4.8 mmol) phenanthrenequinone in the presence of glacial acetic acid (80 mL) for 2 h. The crude product was recrystallized from ethyl acetate to give 1.34 g (80%) of pale-yellow crystals, C_20_H_12_N_2_, 280.33 g/mol, m.p. 224–225 °C lit. 224–226 °C [61].

^1^H NMR (400 MHz, CDCl_3_) δ (ppm): 9.34 (d, *^3^J_H_*_,*H*_ = 8 Hz, 2H), 9.32 (d, *^3^J_H_*_,*H*_ = 8 Hz, 2H), 8.50–8.48 (d, *^3^J_H_*_,*H*_ = 8 Hz, 2H), 8.27–8.24 (m, 2H), 7.870–7.65 (m, 4H).

^13^C NMR (200 MHz, CDCl_3_) δ (ppm): 141.8, 141.3, 132.0, 130.7, 130.2, 129.3, 128.9, 128.0, 126.5, 122,9.

##### DBPh2: 11-methyldibenzo[a,c]phenazine

DBPh2 was obtained in an analogous way to the DBPh1 compound, using 4-methyl-*o*-phenylenediamine instead of 1,2-diaminobenzene. The crude product was recrystallized from ethyl acetate to give 1.17 g (83%) of pale-yellow crystals, C_21_H_14_N_2_, 294.30 g/mol, m.p. 220–222 °C lit. 220–221 °C [61].

^1^H NMR (400 MHz, CDCl_3_) δ (ppm): 9.32–9.29 (m, 2H), 8.50, 8.48 (d, *^3^J_H**,**H_* = 8 Hz, 2H), 8.15–8.13 (d, *^3^J_H_*_,*H*_ = 8 Hz, 2H), 8.02 (s, 1H), 7.73–7.59 (m, 5H), 2.60 (s, 3H).

^13^C NMR (200 MHz, CDCl_3_) δ (ppm): 142.23, 142.16, 141.68, 140.74, 140.45, 132.46, 132.03, 131.84, 130.37, 130.32, 130.19, 130.05 128.09, 127.95, 127.89, 127.87, 126.23, 126.09, 122.89, 22.07.

##### DBPh3: 11-methoxydibenzo[a,c]phenazine

DBPh3 was obtained in an analogous way to the DBPh1 compound, using 4-methoxy-*o*-phenylenediamine dihydrochloride instead of 1,2-diaminobenzene. The crude product was recrystallized from ethyl acetate to give 1.17 g (91%) of pale-yellow crystals, C_21_H_14_N_2_O, 310.35 g/mol, m.p. 200–202 °C lit. 201–203 °C [58].

^1^H NMR (400 MHz, CDCl_3_) δ (ppm): 9.46-9.44 (d, *^3^J_H_*_,*H*_ =8 Hz, 1H), 9.37-9.35 (dd, *^3^J_H**,**H_* = 8 Hz, 1H), 8.60–8.57 (t, 2H), 8.23–8.21 (d, *^3^J_H_*_,*H*_ =8 Hz, 1H), 7.84–7.73 (m, 5H), 7.55–7.52 (dd, *^3^J_H_*_,*H*_ =8 Hz, 1H), 4.09 (s, 3H).

^13^C NMR (200 MHz, CDCl_3_) δ (ppm): 161.24, 143.09, 141.54, 140.28, 138.82, 132.22, 131.38, 130.66, 130.44, 130.37, 129.77, 129.65, 127.94, 127.87, 126.34, 125.74, 124.42, 122.95, 122.86, 105.42, 56.00.

##### DBPh4: Dibenzo[a,c]phenazine-2-carboxylic Acid

DBPh4 was obtained in an analogous way to the DBPh1 compound, using 3,4-diaminobenzoic acid instead of 1,2-diaminobenzene. The crude product was recrystallized from ethyl acetate to give 1.44 g (93%) of pale-yellow crystals, C_21_H_12_N_2_O_2_, 324.33 g/mol, m.p. 310–312 °C.

^1^H NMR (400 MHz, DMSO-*d*_6_) δ (ppm): 9.26-9.24 (d, *^3^J_H_*_,*H*_ =8 Hz, 2H), 8.82 (s, 1H), 8.80–8.78 (d, *^3^J_H_*_,*H*_ =8 Hz, 2H), 8.38–8.37 (t, 2H), 7.93–7.89 (m, 2H) 7.84–7.80 (t, 2H).

^13^C NMR (200 MHz, DMSO-*d*_6_) δ (ppm): 167.18, 143.54, 143.48, 143.07, 141.10, 132.82, 132.45, 132.15, 131.93, 131.72, 131.57, 129.96, 129.91, 129.54, 128.93, 128.90, 126.45, 126.25, 124.20, 124.16.

##### DBPh5: Dibenzo[a,c]phenazine-2-carboxylic Acid Methyl Ester

DBPh5 was obtained in an analogous way to the DBPh1 compound, using methyl 3,4-diaminobenzoate instead of 1,2-diaminobenzene. The crude product was recrystallized from ethyl acetate to give 1.46 g (90%) of pale-yellow crystals, C_22_H_14_N_2_O_2_, 338.36 g/mol, m.p. 296–298 °C.

^1^H NMR (400 MHz, DMSO-*d*_6_) δ (ppm): 9.32–9.30 (d, ^*3*^*J_H_*_,*H*_ = 8 Hz, 2H), 8.91 (s, 1H), 8.91–8.83 (d, ^*3*^*J_H_*_,*H*_ = 8 Hz, 2H), 8.48–8.40 (m, 2H), 7.97–7.93 (m, 2H), 7.93–7.85 (m, 2H), 4.02 (s, 3H).

^13^C NMR—the spectrum was not recorded due to too-low solubility.

##### DBPh6: 11-benzoyldibenzo[a,c]phenazine

DBPh6 was obtained in an analogous way to the DBPh1 compound, using 3,4-diamonobenzophenone instead of 1,2-diaminobenzene. The crude product was recrystallized from ethyl acetate to give 1.46 g (84%) of pale-yellow crystals, C_27_H_16_N_2_O, 384.43 g/mol, m.p. 244–246 °C lit. 245–247 °C [62].

^1^H NMR (400 MHz, CDCl_3_) δ (ppm): 9.46–9.44 (d, ^*3*^*J_H_*_,*H*_ = 8 Hz, 1H), 9.38–9.36 (d, ^*3*^*J_H_*_,*H*_ = 8 Hz, 1H), 8.71 (d, ^*3*^*J_H_*_,*H*_ = 8 Hz, 1H), 8.60–8.58 (d, ^*3*^*J_H_*_,*H*_ = 8 Hz, 2H), 8.47–8.45 (d, ^*3*^*J_H_*_,*H*_ = 8 Hz, 1H), 8.38–8.35 (dd, *^3^J_H_*_,*H*_ = 8 Hz, 1H), 8.00–7.98 (m, 2H), 7.88–7.74 (m, 5H), 7.73–7.71 (d, ^*3*^*J_H_*_,*H*_ = 8 Hz, 1H), 7.63–7.59 (t, 2H). 

^13^C NMR (200 MHz, CDCl_3_) δ (ppm): 195.98, 143.90, 143.78, 143.51, 141.09, 137.98, 137.40, 132.92, 132.82, 132.53, 132.22, 131.00, 130.77, 130.21, 129.99, 129.95, 12941, 128.57, 128.14, 126.72, 126.39, 123.03.

##### DBPh7: 11-carbonitriledibenzo[a,c]phenazine

DBPh7 was obtained in an analogous way to the DBPh1 compound, using 3,4-diaminobenzonitrile instead of 1,2-diaminobenzene. The crude product was recrystallized from ethyl acetate to give 1.20 g (82%) of pale-yellow crystals, C_21_H_11_N_3_, 305.33 g/mol, m.p. 274–275 °C lit. 275–276 °C [58].

^1^H NMR (400 MHz, DMSO-d_6_) δ (ppm): 9.30–9.25 (m, 2H), 8.97 (s, 1H), 8.85–8.83 (d, ^*3*^*J_H_*_,*H*_ = 8 Hz, 2H), 8.50–8.48 (d, ^*3*^*J_H_*_,*H*_ = 8 Hz, 1H), 8.27–8.25 (d, ^*3*^*J_H_*_,*H*_ = 8 Hz, 1H), 7.98–7.93 (m 2H), 7.89–7.84 (m, 2H).

^13^C NMR (200 MHz, DMSO-*d*_6_) δ (ppm): 143.80, 143.20, 140,84, 136.07, 132.74, 132.45, 132.38, 132.16, 131.29, 131.22, 129.38, 129.08, 126.07, 126.42, 124.35, 113.02.

##### DBPh8: 11-bromodibenzo[a,c]phenazine

DBPh7 was obtained in an analogous way to the DBPh1 compound, using 4-bromo-1,2-diaminobenzene instead of 1,2-diaminobenzene. The crude product was recrystallized from ethyl acetate to give 1.50 g (87%) of light-yellow crystals, C_20_H_11_BrN_2_, 359.22 g/mol, m.p. 274–275 °C lit. 274–276 °C [58].

^1^H NMR (400 MHz, CDCl_3_) δ (ppm): 9.38–9.35 (m, 2H), 8,59–8.57 (d, ^*3*^*J_H_*_,*H*_ = 8 Hz, 2H), 8.53 (d, 1H), 8.21–8.19 (d, ^*3*^*J_H_*_,*H*_ = 8 Hz, 1H), 7.94–7.91 (m, 1H), 7.85–7.74 (m, 4H).

^13^C NMR (200 MHz, CDCl_3_) δ (ppm): 133.7, 132.3, 132.26, 131.2, 131.0, 130.9, 130.2, 128.2, 128.1, 126.6, 126.6, 123.0, 123.0.

##### DBPh9: 11-iododibenzo[a,c]phenazine

11-iododibenzo[a,c]phenazine (DBPh8) was obtained in a two-step reaction. 

Step 1: synthesis of 4-iodo-1,2-diaminobenzene

To a stirred solution of tin(II) chloride (4.6 g) in concentrated hydrochloric acid (35.4 mL), 1.5 g (5.7 mmol) of 4-iodo-2-nitroaniline was added in portions over a period of approx. 40 min. The reaction mixture was held at 65–70 °C for 1 h. Then, it was cooled to 0–5 °C and left for 16 h to crystallize the tin complex. The complex was dissolved in water, and then a sodium hydroxide solution (5.9 g in 14.7 mL of water) was added with intensive stirring. The reaction temperature was kept below 20 °C. The separated product was washed with water to neutral pH and recrystallized from cyclohexane to obtain 1.07 g (81%) of pale-yellow crystals, C_6_H_7_IN_2_, 234.039 g/mol, m.p. 76–78 °C lit. 75–77 °C [63].

Step 2: synthesis of 11-iodoodibenzo[a,c]phenazine

The compound 11-iododibenzo[a,c]phenazine was obtained by refluxing 1.12 g (4.8 mmol) 4-iodo-1,2-diaminobenzene with 1.0 g (4.8 mmol) phenanthrenequinone in the presence of glacial acetic acid (80 mL) for 1 h. The crude product was recrystallized from ethyl acetate to obtain 1.71 g (88%) of pale-yellow crystals, C_20_H_11_IN_2_, 406.22 g/mol, m.p. 231–233 °C lit. 232–234 °C [58].

^1^H NMR (400 MHz, CDCl_3_) δ (ppm): 9.32–9.28 (m, 2H), 8,72–8.707 (d, ^*3*^*J_H_*_,*H*_ = 8 Hz, 1H), 8.52-8.50 (d, *^3^J_H_*_,*H*_ = 8 Hz, 2H), 8.03–8.75 (m, 2H), 7.77–7.66 (m, 4H).

^13^C NMR (200 MHz, CDCl_3_) δ (ppm): 133.8, 132.2, 132.2, 131.1, 131.0, 130.7, 130.1, 128.3, 128.0, 126.5, 126.4, 123.0, 123.1.

### 2.3. Methods

A Bruker AscendTM 400 NMR spectrophotometer (Billerica, MA, USA) was used to obtain NMR spectra. They were recorded in deuterated chloroform (CDCl_3_) for the compounds DBPh1, DBPh2, DBPh3, DBPh6, DBPh8, and DBPh9, or in deuterated dimethyl sulfoxide (DMSO-*d*_6_) for the compounds DBPh4, DBPh5, and DBPh7. Spectra of the selected compounds carried out under photoreduction conditions were recorded in deuterated acetonitrile (CD_3_CN).

Steady-state absorption measurements were performed on a Shimadzu UV-Vis Multispec-1501 spectrophotometer (Kioto, Japan) in ethyl acetate.

Polymerization kinetics were studied using the microcalorimetric method [28,64]. The tested photocurable compositions consisted of 1-methyl-2-pyrrolidone (MP; 0.1 mL) and trimethylolpropane triacrylate monomer (TMPTA; 0.9 g). The chain reaction was photoinitiated using two-component donor–acceptor systems. The role of an electron acceptor (photoinitiator) was played by synthesized dyes with a concentration of 2.00 × 10^−4^–5.4 × 10^−4^ M (with regard to molar absorptivity). The co-initiators (an electron donors) were PhTAA, PhAA, DMA, and MBO with a concentration of 0.1 M. The photocurable composition was placed in a mold prepared from a Teflon ring (diameter 10 mm × thickness 3 mm) protected on one side with a glass plate using a Pasteur pipette (Paris, France). A thermocouple (RTD Thermometer Delta OHM HD 2107.1, Atlanta, GA, USA) was used as a temperature change sensor, which was immersed in the tested sample. After a 10 s delay, the polymerizing mixture was irradiated from the bottom with an LED dental lamp (Cromalux 75 Mega Phisik Dental, Rastatt, Germany), emitting blue light in the range of 390–500 nm, with an intensity of 20 mW/cm^2^. A Coherent Field Master meter (Minneapolis, MN, USA) was used to set the appropriate radiation intensity falling on the sample located at a constant distance from the light source. Temperature changes were recorded every 1 s for 1 min with a recorder (Delta OHM HD 40.1; reading accuracy ±0.1°) with three repetitions for each sample.

The effectiveness of the tested systems for initiating the triacrylate chain reaction was checked in comparison to camphorquinone (CQ)—a commercially used photoinitiator. Its concentration in the tested photocurable compositions was 0.675 M.

## 3. Results and Discussion

### 3.1. Selection and Modification of the Compounds Tested

Nine compounds based on the dibenzo[a,c]phenazine (DBPh1–DBPh9) skeleton were synthesized. This structure was chosen because it is highly stiffened due to having five fused aromatic rings. Stiffening of the molecule prevents rotation of the benzene rings and blocks the possibility of isomerization, as is the case with phenyl rings separated by a polymethine bridge. Thanks to this, excited-state deactivation channels are eliminated, which is extremely important when using these compounds in photochemical processes [34,43,65]. Moreover, the biological activity of dibenzo[a,c]phenazine was also documented. This compound, due to the presence of nitrogen-based heterocyclic fragments in its structure, is used to design compounds with pharmacological, [66,67] especially anticancer, [68] activity. 

The designed compounds also had to exhibit appropriate spectroscopic properties, mainly absorption. Therefore, the structure of dibenzo[a,c]phenazine was modified by introducing electron-donating (DBPh2, DBPh3) and electron-withdrawing (DBPh4–DBPh7) substituents, and heavy atoms (DBPh8–DBPh9) at position 11, which made it possible to obtain potentially effective radical polymerization initiators active in the visible light region. 

### 3.2. Photophysical Data

**Figure 3:**
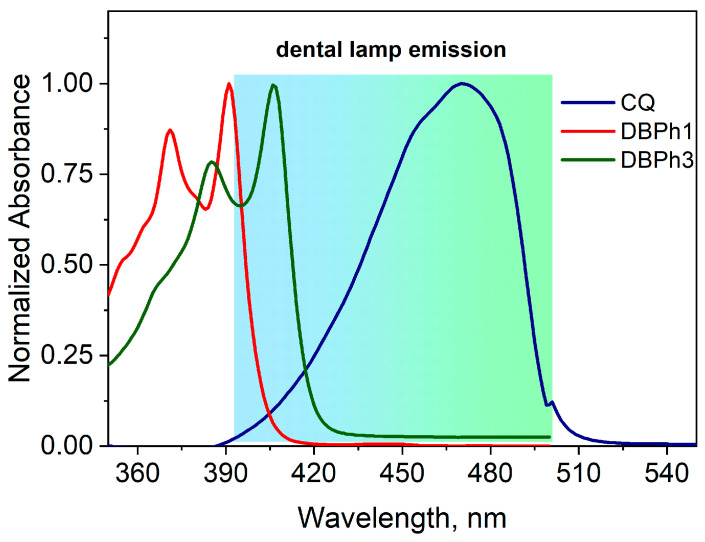
Normalized electronic absorption spectra of DBPh1, DBPh3, and CQ in ethyl acetate. The background color in the figure indicates the light emission area of the Cromalux 75 lamp.

**Figure 4:**
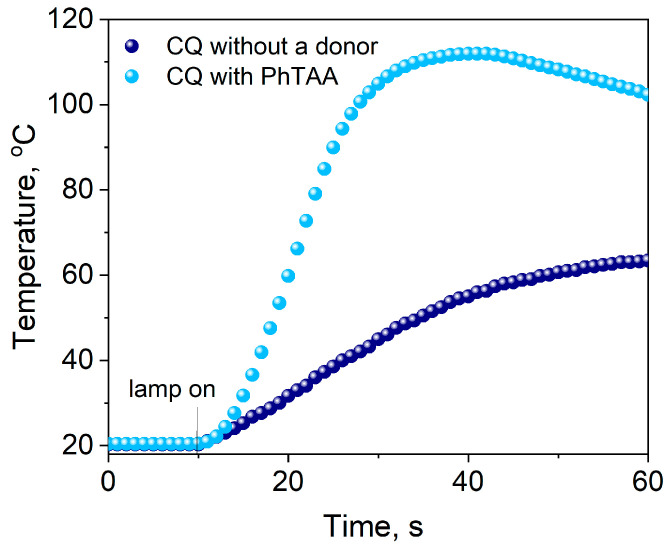
Kinetic curves of TMPTA polymerization photoinitiated by camphorquinone (CQ) coupled with a co-initiator—thiophenoxyacetic acid (0.1 M)—and without an electron donor. The light intensity emitted by the dental lamp was 20 mW cm^−2^.

**Figure 5:**
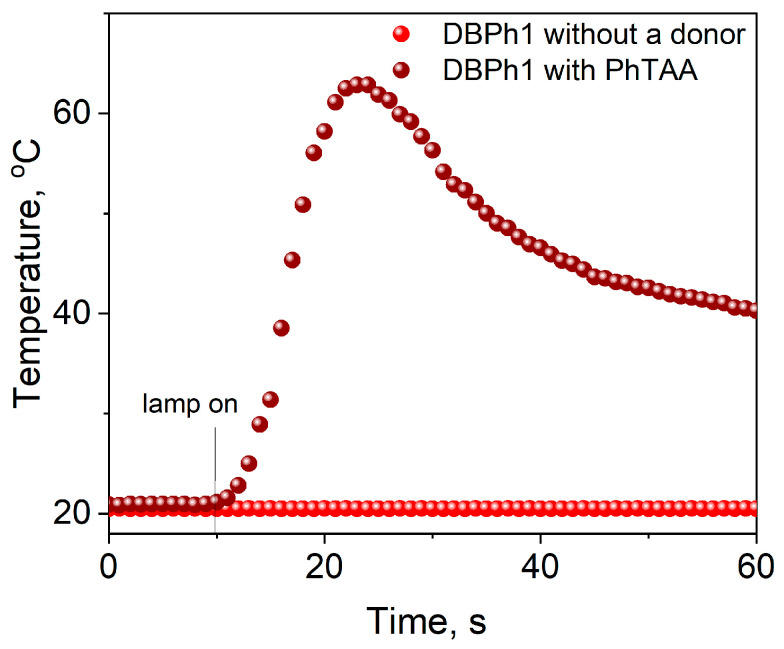
Kinetic curves of TMPTA polymerization photoinitiated by dibenzo[a,c]phenazine (DBPh1) coupled with a co-initiator—thiophenoxyacetic acid (0.1 M)—and without an electron donor. The light intensity emitted by the dental lamp was 20 mW cm^−2^.

Electronic absorption spectra of the tested compounds (DBPh1–DBPh9) recorded in ethyl acetate are presented in Figure 3, Figure 4 and Figure 5. Basic spectroscopic data including the wavelength at the absorption maximum (λ_max_) and molar absorptivity are collected in Table 1.

The electronic absorption spectra of the synthesized dyes are characterized by more than one absorption maximum, which results from the presence of several fused aromatic rings in the molecule. Unsubstituted dibenzo[a,c]phenazine (DBPh1) has an absorption maximum at 395 nm. The introduction of a substituent into the parent molecule causes the absorption band to shift towards longer wavelengths, so that the dyes DBPh3 and DBPh5–DBPh9 absorb in the visible region, while the absorption bands of the remaining ones (DBPh2 and DBPh4) overlap the visible region. The data collected in Table 1 prove that the presence of electron-donating (–CH_3_, –OCH_3_) and electron-accepting (COOH, COOCH_3_, –C(O)C_6_H_5_, –CN) substituents and heavy atoms (Br, I) in the structure of the dye, compared to the parent compound (DBPh1), shift the absorption bands to the red by approximately 3–11 nm (bathochromic effect) without changing their shape.

The introduction of an electron-donating substituent into the structure of dibenzo[a,c]phenazine (Figure S1), i.e., a methyl or methoxy group, causes a red shift of approximately 3 nm or 9 nm, respectively (DBPh1 vs. DBPh2, DBPh1 vs. DBPh3). These substituents can donate a lone electron pair to the system of conjugated double bonds of the aromatic ring. As a result, a partial charge transfer occurs from the oxygen atom (–OCH_3_) or carbon atom (–CH_3_) to the phenyl ring. Therefore, DBPh2 and DBPh3 absorb visible light more effectively compared to DBPh1. A similar bathochromic effect in relation to the long-wavelength absorption band is observed in the case of the introduction of electron-withdrawing substituents, such as carboxyl, ester, benzoyl, and cyano groups, which have the ability to accept an electron from the aromatic ring (DBPh1 vs. DBPh4, DBPh5, DBPh6, and DBPh7—Figure S2).

The introduction of a heavy atom, i.e., bromine or iodine, into the structure of dibenzo[a,c]phenazine also causes a shift of the absorption maximum towards longer wavelengths. It is approximately 7–10 nm (DBPh1 vs. DBPh8 and DBPh9—Figure S3).

The consequence of modifying the structure of dibenzo[a,c]phenazine is the shift of the absorption bands toward longer wavelengths and their overlap with the visible area. This guarantees effective absorption of radiation emitted by the dental lamp (390 nm–500 nm) used in the photopolymerization process.

Camphorquinone, which is a photoinitiator commonly used in dentistry, absorbs radiation in the range of 200–300 nm (ππ* transition) and 400–500 nm (nπ* transition) [69]. The presence of this long-wavelength absorption band makes this compound widely used as a photoinitiator of photocurable dental materials [44,70]. However, absorption in the visible region is responsible for its intense yellow color. This, in turn, affects the aesthetics of the reconstruction of hard dental tissues and the quality of the final product. Figure 3 compares the position of the absorption bands of the tested dyes and camphorquinone in relation to the radiation emitted by the dental lamp.

In the emission area of the Cromalux 75 dental lamp, there is an absorption band of camphorquinone with a maximum at 472 nm. The longer-wavelength band position of the parent compound (DBPh1) occurs at 395 nm. However, its arm overlaps the lamp emission area. The introduction of a methoxy group to the structure of dibenzo[a,c]phenazine shifts the maximum absorption band to 405 nm. Additionally, the molar absorption coefficient of the synthesized compounds (DBPh1–DBPh9) for the long-wavelength band ranges from 11,400 to 28,300 M^−1^cm^−1^, while for commercial camphorquinone this parameter at 472 nm is 40 M^−1^cm^−1^. In practice, this means the possibility of lowering the amount of photoinitiator used, and thus, greater savings resulting from the reduced costs of electricity and materials.

### 3.3. Photopolymerization

Commercially available initiating systems used in dentistry are two-component systems consisting of camphorquinone acting as a photoinitiator and a tertiary aromatic amine—a co-initiator. When exposed to a dental lamp, the camphorquinone molecule is excited. As a result of the interaction with the amine molecule in the excited state, the process of electron transfer (PET) occurs from the co-initiator to the photoinitiator [25,43,53]. Then, subsequent reactions take place, i.e., proton transfer, proton transfer and decarboxylation, and others. The free radicals that can start a chain reaction come mainly from the amine. Their generation is determined by the rate of the primary process, i.e., PET. In the case of electron donor–acceptor systems without electrostatic interactions in the ground state, the PET process is controlled by diffusion and, therefore, depends on the distance between the components of the photoredox pair.

Camphorquinone can initiate radical polymerization itself without a co-initiator, but the rate is unsatisfactory and insufficient (*R_p_* = 29.86 μmol s^−1^). The introduction of a second component—a co-initiator, e.g., PhTAA—into the photocurable composition causes a fivefold increase in the rate of this process (*R_p_* = 144.10 μmol s^−1^) (Figure 4). 

In the photocurable compositions tested in this work, the presence of the second component is necessary (Figure 5) because samples without a co-initiator do not photoinitiate the polymerization process (*R_p_* ≈ 2 μmol s^−1^). 

#### 3.3.1. Role of the Photoinitiator

**Figure 6:**
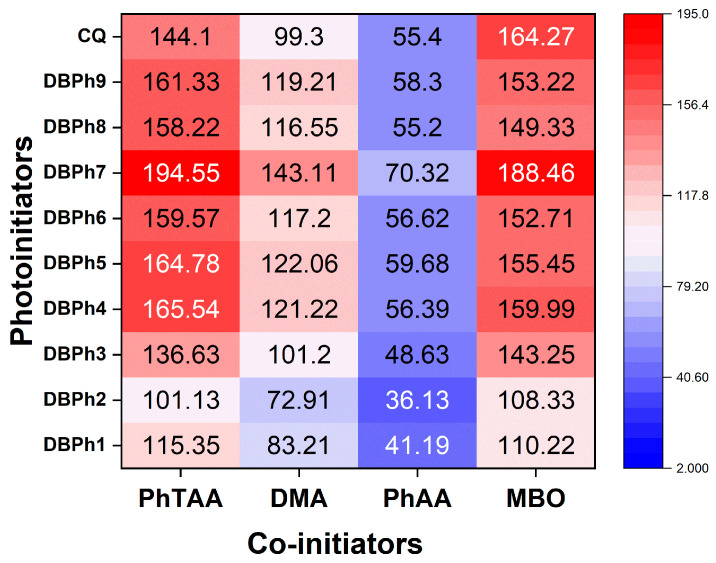
Influence of the type of photoinitiator and co-initiator on the initial rate of TMPTA polymerization (μmol s^−1^) in the form of a heatmap. The light intensity emitted by the dental lamp was 20 mW cm^−2^.

The dominant role of the photoinitiator in the initiating systems is related not only to determining the rate of photopolymerization but also to the influence on the physical and mechanical properties of polymeric materials [71]. Figure 6 shows the influence of the type of photoinitiator and co-initiator on the initial rate of TMPTA polymerization in the form of a heatmap.

Analysis of the data presented in Figure 6 proves that the ability to photoinitiate radical polymerization depends significantly on the structure of the tested dibenzo[a,c]phenazines. The most effective photoinitiators of TMPTA polymerization are compounds containing electron-withdrawing substituents (DBPh4–DBPh7) and heavy atoms (DBPh8 and DBPh9). When designing the structures of these compounds, we took into account previous research results on the heavy-atom effect based on the skeleton of pyridopyrazinoindole, pyrazolo[3,4-b]quinoxaline [72], pyrazoloquinoline [73], quinolineimidazopyridine [74], styrylbenzothiazole [75], naphthoylenebenzimidazolone [76], and quinoxaline[2,3-b]quinoxaline [77]. Chlorine, bromine, or iodine atoms are also often present in the structure of dyes used in photochemistry, e.g., Rose Bengal [78], RBAX [79], or 3-hydroxy-6-fluorone derivatives [80].

**Figure 7:**
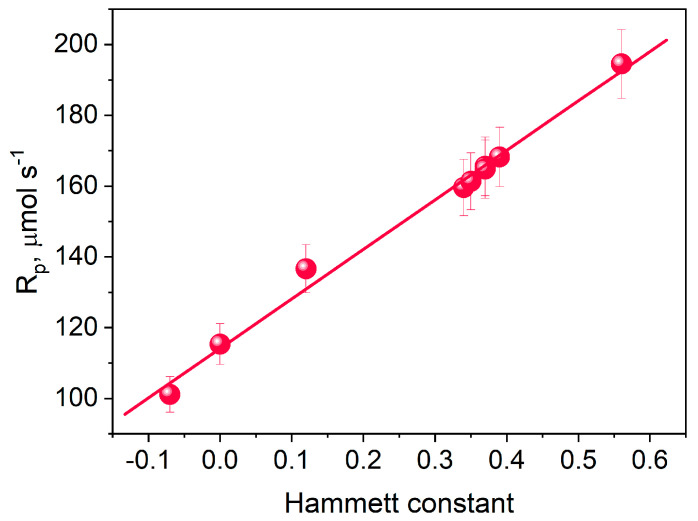
Dependence of Hammett substituent constants on the initial rate of TMPTA photopolymerization.

The substituent effect in the tested photoinitiators was verified using empirically determined Hammett substituent constants (Figure 7) [81]. The data presented in Figure 7 indicates a linear correlation between the Hammett substituent constants and the initial rate of TMPTA polymerization. This means that when designing the structure of dibenzo[a,c]phenazine derivatives, the influence of the substituent on the photoinitiating capacity of the final product can be easily predicted. The choice of research objects resulted from the fact that Hammett substituent constants characterize the different nature of intramolecular interactions of the introduced substituents. Electron-donating substituents, e.g., a methoxy group or a methyl group, have lower values of Hammett substituent constants compared to electron-withdrawing substituents, e.g., a carboxyl, cyano, ester, or benzoyl groups. According to the data presented in Figure 6 and Figure 7, they should reduce the rate of the TMPTA photopolymerization process.

A comparison of the photoinitiating abilities of DBPh7 with derivatives of quinoxaline, indenoquinoxaline, indoloquinoxaline, and quinolinopyridine described in our previous papers [37,64,72,74,82] is presented in Figure S4. Analysis of the initial rates of TMPTA polymerization initiated by the above-mentioned photoinitiators indicates that a system containing dibenzo[a,c]phenazine as a photoinitiator is highly efficient (Table S1).

#### 3.3.2. Role of the Co-Initiator

The heatmap presented in Figure 6 shows that the initial rate of TMPTA polymerization is determined not only by the chemical structure of the photoinitiator but primarily by the type of co-initiator. The choice of electron donors was dictated by the elimination of tertiary amines from the photocurable composition used in commercial products, which are often genetic and cytotoxic agents. [83]. Therefore, phenylthioacetic acid (PhTAA) and phenoxyacetic acid (PhAA) were used as co-initiators instead. For comparison, *N*,*N*-dimethylaniline (DMA) was tested as well.

**Figure 8:**
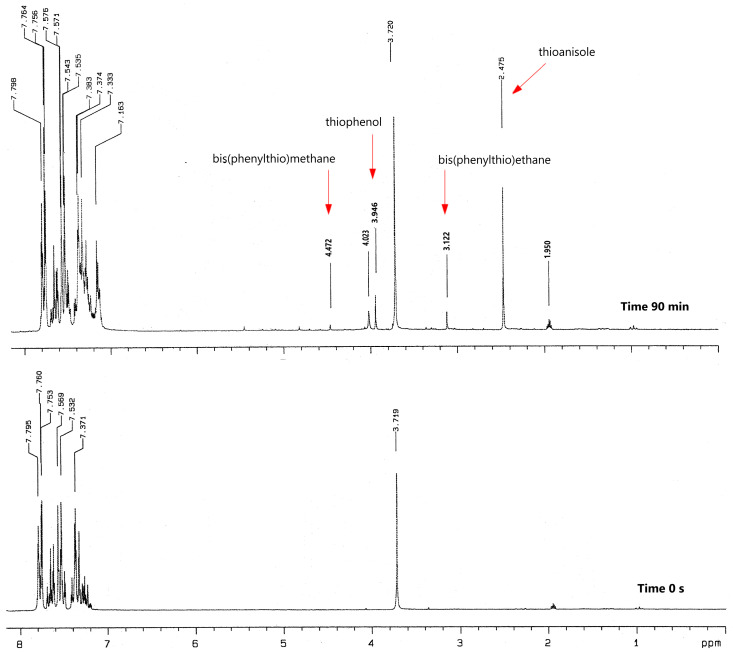
^1^H NMR spectra of a mixture of dibenzo[a,c]phenazine (DBPh1) and (phenylthio)acetic acid in CD_3_CN before and after 90 min of irradiation.

A careful analysis of the presented heatmap (Figure 6) confirms that the PET process is more efficient in photoredox pairs containing electron donors with a sulfur (PhTAA) or a nitrogen (DMA) atom. However, the initial polymerization rate is slower when phenoxyacetic acid is used as a co-initiator. This is related to the type of reactive species formed in secondary reactions following the electron transfer process and their activity in initiating the polymerization reaction. Two radicals, $C_{6}H_{5}S\dot{C}HCOOH\;$ and $C_{6}H_{5}S\dot{C}H_{2}$, are formed from thiophenoxyacetic acid, which are more reactive than the radicals generated from phenoxyacetic acid,${\;C}_{6}H_{5}O\dot{C}HCOOH$ and $C_{6}H_{5}O\dot{C}H_{2}$ [34,82,84]. However, these radicals may undergo secondary reactions producing various products. An analysis of products formed under photoreduction conditions from thiophenoxyacetic acid was carried out. Figure 8 presents the most important fragments of the ^1^H NMR spectra of the sample containing the DBPh1–PhTAA photoredox pair, obtained before and after 90 min of irradiation.

The resulting products were identified by comparison with reference compounds (^1^H NMR spectra included in the ESI file). Table 2 collects the chemical shifts of protons typical for the compounds present in the tested sample.

**Figure 9:**
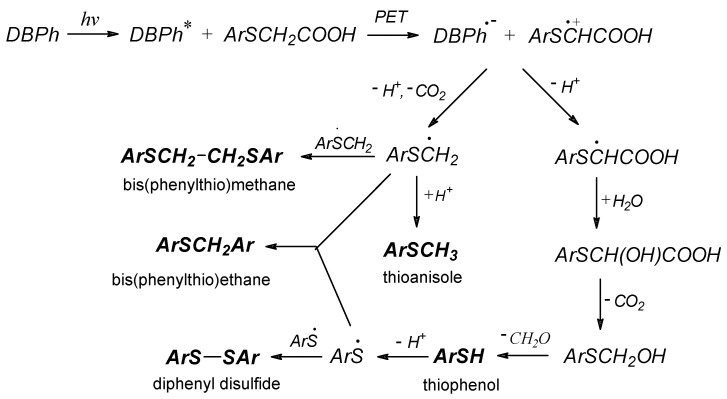
Mechanism of thiophenoxyacetic acid transformation under photoreduction conditions.

The presence of thioanisole, thiophenol, diphenyl disulfide, bis(phenylthio)methane, and bis(phenylthio)ethane was confirmed in the tested samples. Based on the identified compounds, a mechanism for the transformation of thiophenoxyacetic acid under photoreduction conditions was proposed (Figure 9).

However, as a result of the photoreduction of *N*,*N*-dimethylaniline, *N*-methylformanilide, *N*-methylaniline, aniline, and small amounts of other compounds are formed [85]. Using phenoxyacetic acid as an electron donor, anisole, phenol, CO_2_, and 1,2-diphenoxyethane are obtained [86,87].

**Figure 10:**
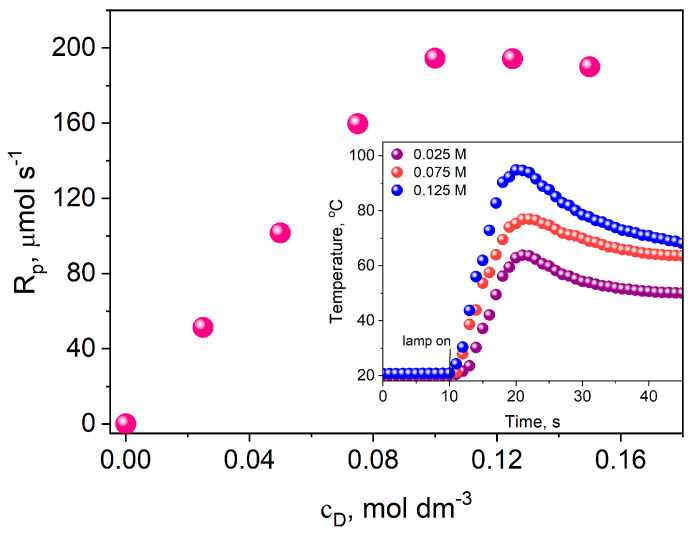
The effect of the concentration of the electron donor on the initial rate of TMPTA polymerization; photoinitiator: DBPh7, co-initiator: PhTAA, light intensity: 20 mW cm^−2^. The pink dots in the figure indicate the initial polymerization rate at a specific electron donor concentration, determined from the kinetic curves shown as an example in the inset.

Moreover, another key parameter influencing the rate of the photopolymerization process is the concentration of the electron donor. Figure 10 shows the effect of electron donor concentration on the initial rate of TMPTA polymerization for an exemplary composition containing DBPh7 as a photoinitiator.

Analysis of the data presented in Figure 10 indicates that the photopolymerization rate rises with an increase in the electron donor concentration up to 0.1 M. Above this value, a further increase in the co-initiator concentration does not affect photopolymerization efficiency; a plateau is observed. This proves that at a concentration of about 0.1 M, the excited state of the photoinitiator is completely quenched, so the optimal concentration is 0.1 M.

#### 3.3.3. Influence of the Light Intensity of the Dental Lamp

The kinetic Equation (1) describing the rate of photoinitiated polymerization shows that an important parameter influencing the rate of this process is the intensity of light absorbed by the photocurable composition.

(1) $$R_{p}=-\frac{d\left[M\right]}{dt}=k_{p}[M]\sqrt{\frac{I_{a}\Phi_{T}}{k_{t}}}$$

where: *M*—molar concentration of monomer, *k_p_*—polymerization rate constant, *I_a_*—absorbed radiation intensity, *Φ_T_*—quantum yield of triplet state formation, and *k_t_*—macroradical termination rate constant

**Figure 11:**
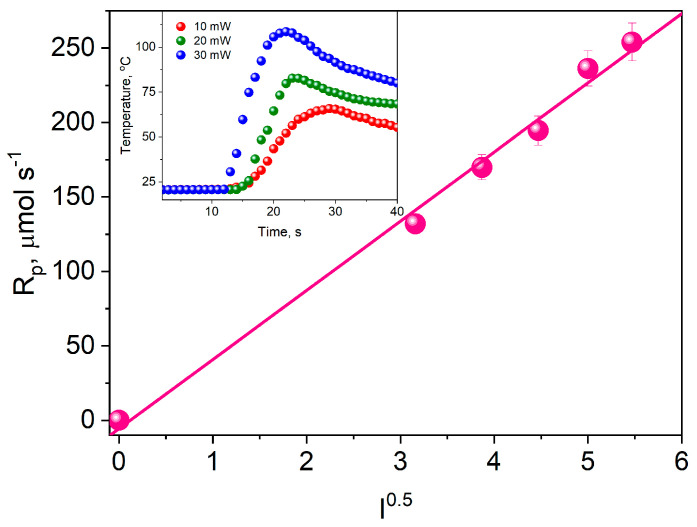
The effect of light intensity on the initial rate of TMPTA photopolymerization initiated by DBPh7 irradiated with a Cromalux 75 dental lamp; co-initiator: PhTAA (c = 0.1 M), the light intensity changed from 10 to 30 mW cm^−2^. The initial polymerization rate at a given light intensity was determined from the kinetic curves shown as an example in the inset.

Figure 11 shows the effect of light intensity on the initial rate of TMPTA photopolymerization initiated by the DBPh7 dye irradiated with a Cromalux 75 dental lamp.

The observed correlation is consistent with Equation (1). The rate of photoinitiated polymerization depends linearly on the square root of the light intensity. Therefore, the chain termination process takes place between two macroradicals. This means that primary radicals are not involved in it.

#### 3.3.4. Photoinitiation via the Hydrogen-Atom Transfer Mechanism

Apart from the PET process, free radicals initiating polymerization can be generated as a result of other photochemical reactions, e.g., hydrogen-atom transfer (HAT) [37,38,39,43]. The excited dye molecule abstracts the hydrogen atom from the co-initiator. This creates radicals that can recombine to form different products. The basic parameter determining the rate of hydrogen-atom transfer is the energy necessary to detach the hydrogen atom from the co-initiator. Amines, alcohols, and thiols are usually used as hydrogen-atom donors [88].

**Figure 12:**
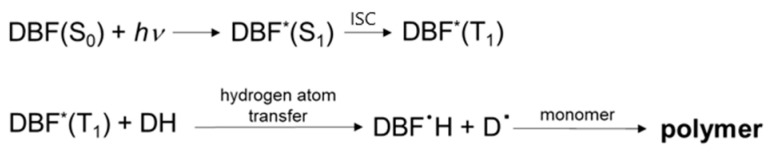
General mechanism for the HAT process for the tested dibenzo[a,c]phenazine derivatives and 2-mercaptobenzoxazole (MBO); DBF—dibenzo[a,c]phenazine, DH—hydrogen-atom donor.

Based on data in the literature [1] and our previous works [34,37], in which MBO was used as the hydrogen-atom donor, a general mechanism for the HAT process can be proposed as shown in Figure 12.

The absorption of a quantum of electromagnetic radiation leads to the excitation of the dye molecule to a singlet excited state. The singlet excited dye is converted to the triplet excited state by an intersystem crossing. This triplet excited dye may abstract the hydrogen atom from 2-mercaptobenzoxazole (MBO) that, in turn, can polymerize acrylic monomers. Thus, two reactive radicals are formed after the HAT process. In the succeeding reactions, these radicals may recombine with each other or other subsequent reactions may occur leading to different products. However, further research is required to properly establish the mechanism. Nevertheless, the developed photoinitiating systems based on dibenzo[a,c]phenazine derivatives and 2-mercaptobenzoxazole (MBO) effectively initiate TMPTA polymerization through the hydrogen-atom transfer mechanism. This is evidenced by the *R_p_* values, which are comparable to the values obtained for photoredox pairs containing PhTAA as an electron donor (Figure 6).

To sum up, it can be stated that dibenzo[a,c]phenazine derivatives can be used as visible light photoinitiators in systems containing both electron donors and hydrogen-atom donors as co-initiators. Radicals initiating TMPTA polymerization in the tested systems may be formed by a mechanism of intermolecular electron transfer, followed by a transfer of protons between the components of the radical ion pair or decarboxylation, depending on the polarity of the solvent, or by hydrogen-atom transfer from 2-mercaptobenzoxazole in the case of hydrogen-atom-transfer photoinitiators.

#### 3.3.5. Comparative Experiments with a Commercial Photoinitiator

**Figure 13:**
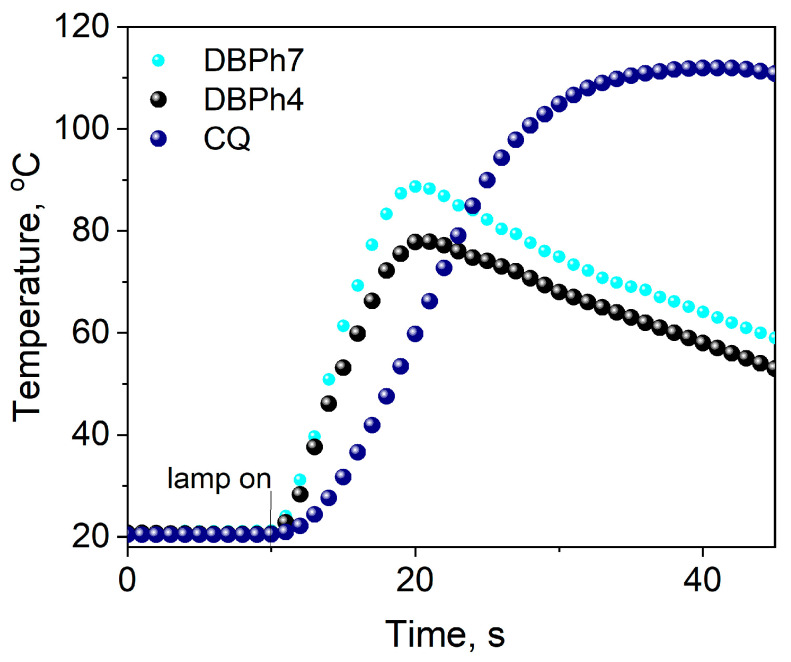
Comparison of the photoinitiating abilities of systems containing dibenzo[a,c]phenazine-2-carboxylic acid (DBPh4) and 11-carbonitriledibenzo[a,c]phenazine (DBPh7) as photoinitiators to camphorquinone (CQ). The tested photoredox pairs have the same electron donor, (phenylthio)acetic acid (PhTAA), at a concentration of 0.1 M; the light intensity of the dental lamp was 20 mW cm^−2^.

To verify the photoinitiating abilities of the tested systems, experiments were performed using a commercial photoinitiator. The comparative samples included photoredox pairs (synthesized dibenzo[a,c]phenazine derivative (DBPh1–DBPh9)—co-initiator) and a CQ—co-initiator (Figure 6 and Figure 13). In all experiments, the number of photons absorbed by the obtained dyes and CQ was the same. As research has shown, the photopolymerization process is significantly influenced by factors that include not only the appropriate selection of the photoinitiator but also its concentration, which should be selected to ensure complete absorption of the light emitted by the dental lamp. In the tested systems, the concentration of photoinitiators (DBPh1–DBPh9) ranged from 2.0 × 10^−4^ to 5.4 × 10^−4^ M, depending on the molar absorption coefficient. However, the concentration of the commercial photoinitiator camphorquinone in analogous tests was 0.675 M. Thanks to this, in the compositions we have developed, it is possible to obtain thick polymer layers (3 mm) without the remains of unreacted photoinitiator, and products formed during its decomposition. Moreover, a too-high concentration of the photoinitiator in dental composites is undesirable because it also affects the aesthetics of the restoration and the quality of the final product. The presence of unreacted camphorquinone that remains in the composite may generate a yellow color.

The data presented in Figure 6 and Figure 13 indicate that the initial rates of TMPTA polymerization for systems containing DBPh3–DBPh9 are higher or comparable to those obtained for the commercial photoinitiator—CQ. In the case of the tested photoinitiators, a hard polymer glass is obtained after approximately 20 s of irradiation (Figure 13). Moreover, the lower temperature increase during polymerization initiated by the dibenzo[a,c]phenazine derivative—thiophenoxyacetic acid system compared to CQ is essential for their potential application in dentistry.

## 4. Conclusions

In this work, dibenzo[a,c]phenazine was synthesized, which was then modified to obtain eight derivatives containing electron-donating and electron-withdrawing substituents and heavy atoms. The new compounds were characterized structurally by ^1^H and ^13^C NMR techniques. They were used in two-component photoinitiating systems in which the second component was an acetic acid derivative. These systems effectively initiate TMPTA polymerization upon irradiation with visible light, induced by a dental lamp through both electron transfer or hydrogen-atom transfer mechanisms. Good photoinitiation abilities have been confirmed by comparative tests with systems containing the photoinitiator commonly used in dentistry—camphorquinone (CQ). Six of the designed dyes, DBPh4–DBPh9, show higher values of the initial rate of acrylates polymerization compared to CQ. In the case of the tested photoinitiators, a hard polymer glaze is obtained after approximately 20 s of irradiation. Moreover, the concentration of the commercial photoinitiator camphorquinone in the photocurable composition is several thousand times higher than that of the tested compounds. Thanks to this, the compositions we test make it possible to obtain thick polymer layers (3 mm) without the remains of unreacted photoinitiator, and products formed during its decomposition. This also affects the aesthetics of the reconstruction and the quality of the final product. In the case of newly developed photoredox systems, much smaller amounts of photoinitiator can be used without slowing down the rate of obtaining polymeric materials.

## Figures and Tables

**Table 1:** Structure and absorption properties of the tested compounds measured in ethyl acetate; λ_max_—wavelength at absorption maximum in nm, ε—molar absorption coefficient in M^−1^cm^−1^.

Dye	Structure	λ_max_	ε	Dye	Structure	λ_max_	ε
DBPh1	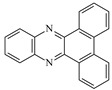	375 395	9700 11,400	DBPh6	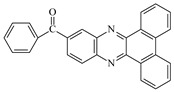	385 406	14,900 20,500
DBPh2	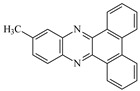	378 398	11,500 18,300	DBPh7	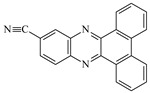	393 413	19,400 20,600
DBPh3	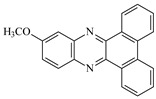	384 405	15,600 21,100	DBPh8	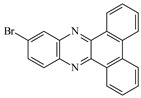	382 403	20,500 25,600
DBPh4	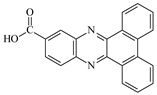	377 399	23,500 27,000	DBPh9	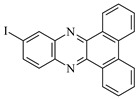	385 406	20,400 26,900
DBPh5	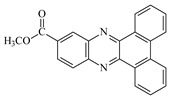	383 404	19,200 28,300	CQ	* 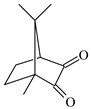 *	472	40

**Table 2:** Values of characteristic chemical shifts of protons for products formed under photoreduction conditions from thiophenoxyacetic acid (^1^H NMR).

Compound	Structure	σ, ppm
thioanisole	C_6_H_5_SCH_3_	2.47
thiophenol	C_6_H_5_SH	3.95
Diphenyl disulfide	C_6_H_5_S-SC_6_H_5_	-
bis(phenylthio)methane	C_6_H_5_S-CH_2_-SC_6_H_5_	4.47
bis(phenylthio)ethane	C_6_H_5_S-CH_2_-CH_2_-SC_6_H_5_	3.12

## Data Availability

The data presented in this study are available on request from the corresponding author due to privacy.

## Supplementary Materials

The following supporting information can be downloaded at https://www.mdpi.com/article/10.3390/ma17112597/s1. ^1^H NMR, ^13^C NMR spectra; Figures S1–S3: UV–visible spectra for all tested compounds; Figure S4: Kinetic curves of photoinitiated polymerization of TMPTA initiated by a photoredox pair containing different initiators; Table S1: Initial rate of photoinitiated free radical polymerization of TMPTA initiated by photoredox pairs containing different photoinitiators.
