# Supplementary material for: Highly Efficient Photoinitiation Systems Based on Dibenzo[a,c]phenazine Sensitivity to Visible Light for Dentistry
Source: Materials (Basel). 2024 May 28;17(11):2597. doi: 10.3390/ma17112597 (PMC11173425; doi:10.3390/ma17112597)
Supplement: Supplementary file 1 [file materials-17-02597-s001.zip › materials-3007577-supplementary.pdf]

Supporting information

Highly Efficient Photoinitiation Systems Based on Dibenzo[a,c]phenazine Sensitivity to Visible Light for Dentistry

Ilona Pyszka * and Beata Jędrzejewska *

Faculty of Chemical Technology and Engineering, Bydgoszcz University of Science and Technology, ul. Seminaryjna 3, 85-326 Bydgoszcz, Poland
* Correspondence: ilona.pyszka@pbs.edu.pl (I.P.); beata@pbs.edu.pl (B.J.);
Tel.: +48-52-374-9039 (I.P.); +48-52-374-9046 (B.J.)

**Table of contents**	**Page**
^{1}H and ^{13}C NMR spectra…………………………………………………………………………...	2
^{1}H NMR spectra of samples containing standards of products formed from (phenylthio)acetic acid (PhTAA) under photoreduction conditions ..…………………………..	19
Normalized electronic absorption spectra of the dyes differing in the electron-releasing substituent (DBPh1 vs. DBPH 2 and DBPh3) in ethyl acetate – Figure S1 ………………………..	22
Normalized electronic absorption spectra of dyes differing in the electron-withdrawing substituent (DBPh1 vs. DBPh4–DBPh7) in ethyl acetate – Figure S2 ……………………………	22
Normalized electronic absorption spectra of dyes differing in heavy atoms (DBPh1 vs. DBPh8 and DBPh9) in ethyl acetate – Figure S3 ……………………………………………………………	22
Comparison of kinetic curves of TMPTA polymerization photoinitiated by the tested dibenzo[a,c]phenazine (DBPh7) and other dyes coupled with co-initiator – thiophenoxyacetic acid (0.1 M). The light intensity emitted by the dental lamp was 20 mW cm^{-2} – Figure S4 ……	23
Initial rate of TMPTA polymerization photoinitiated by different donor-acceptor couples - Table S1 ………………………………………………………………………………………...	23-24

^{1}H spectrum of **DBPh1**

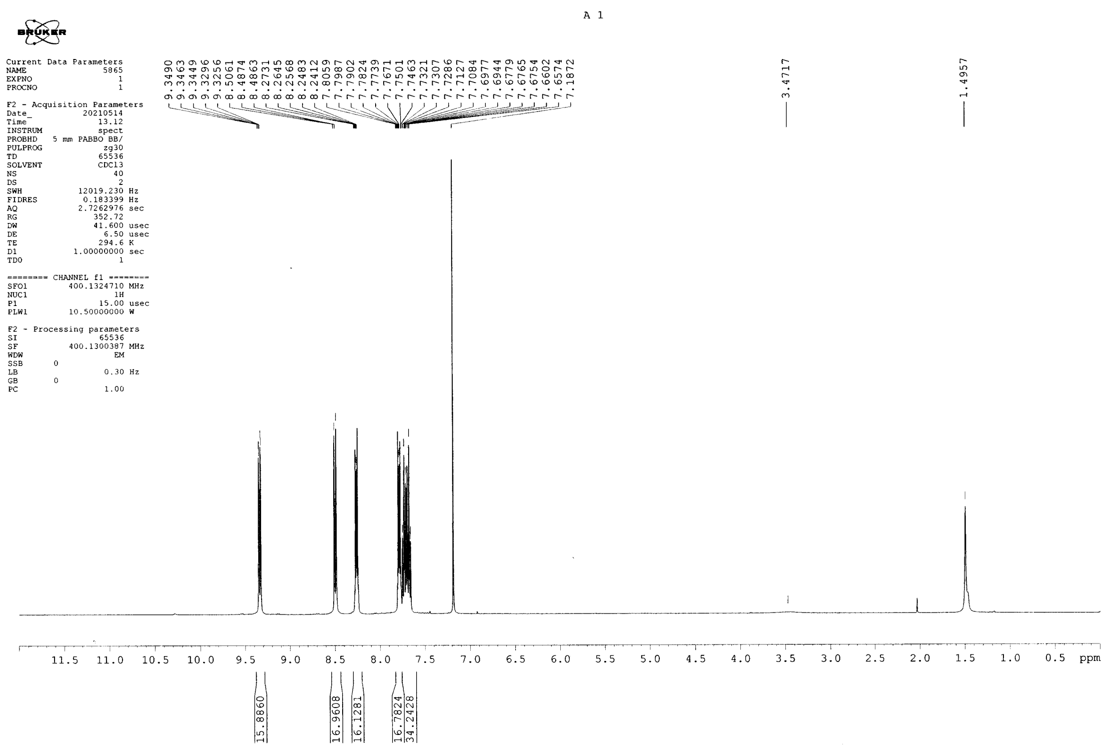

Enlarged spectrum in the range of 6-9 ppm.

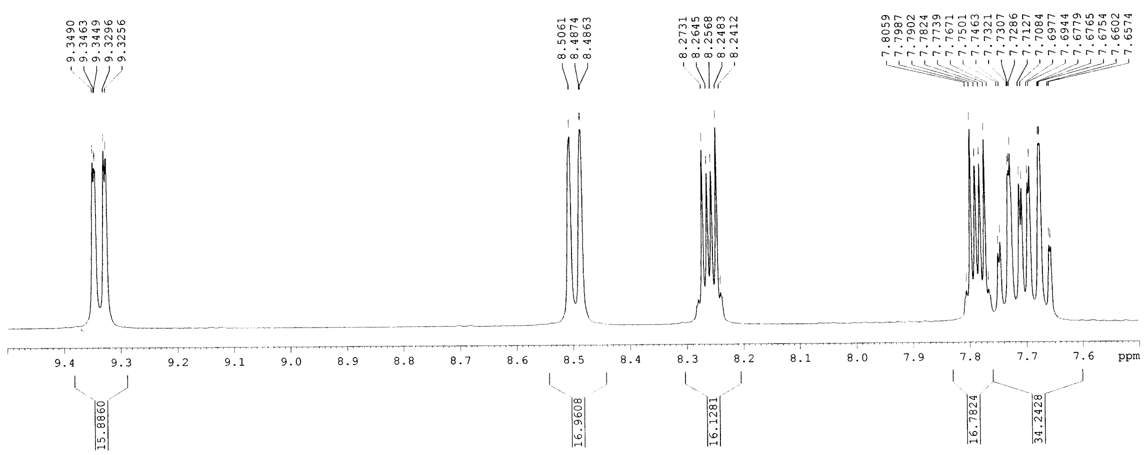

^{13}C spectrum of **DBPh1**

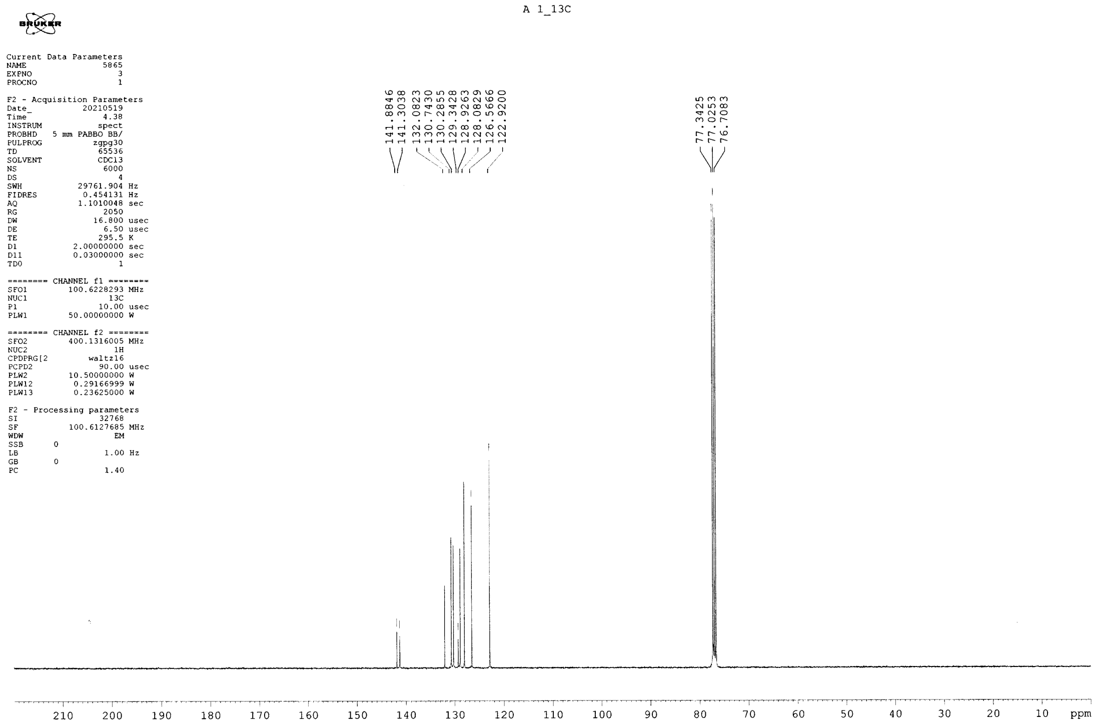

Enlarged spectrum in the range of 120-145 ppm.

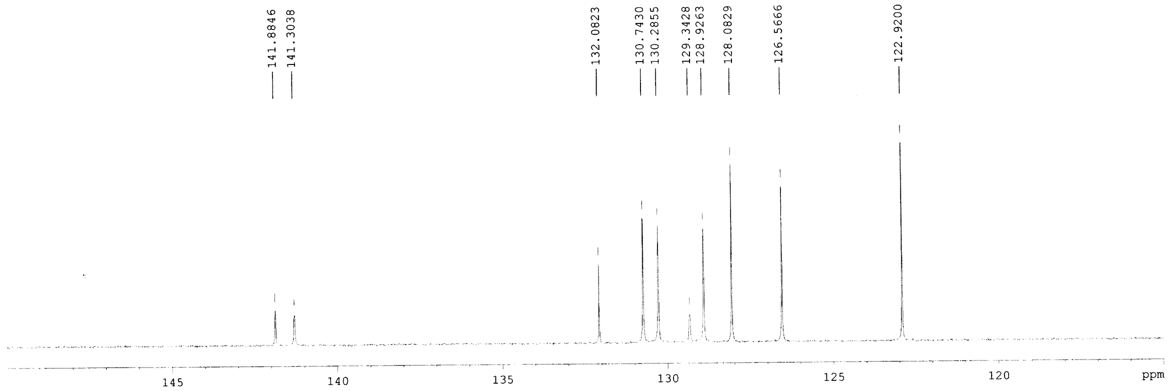

^{1}H spectrum of **DBPh2**

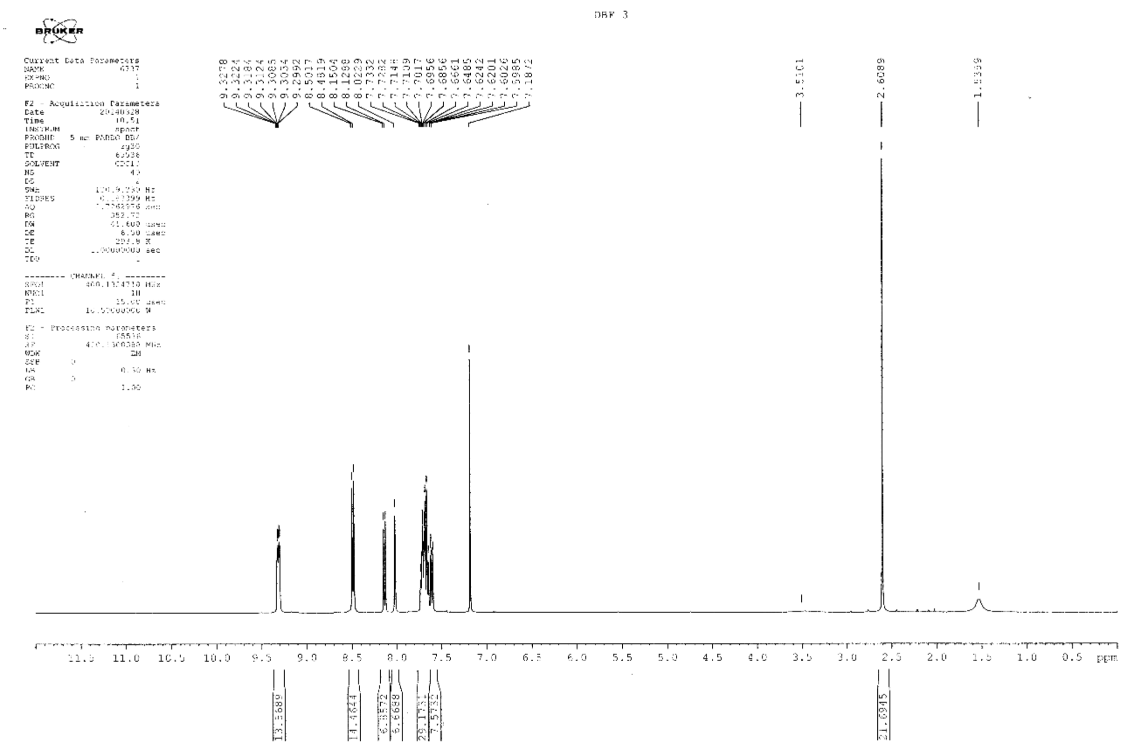

Enlarged spectrum in the range of 7-10 ppm.

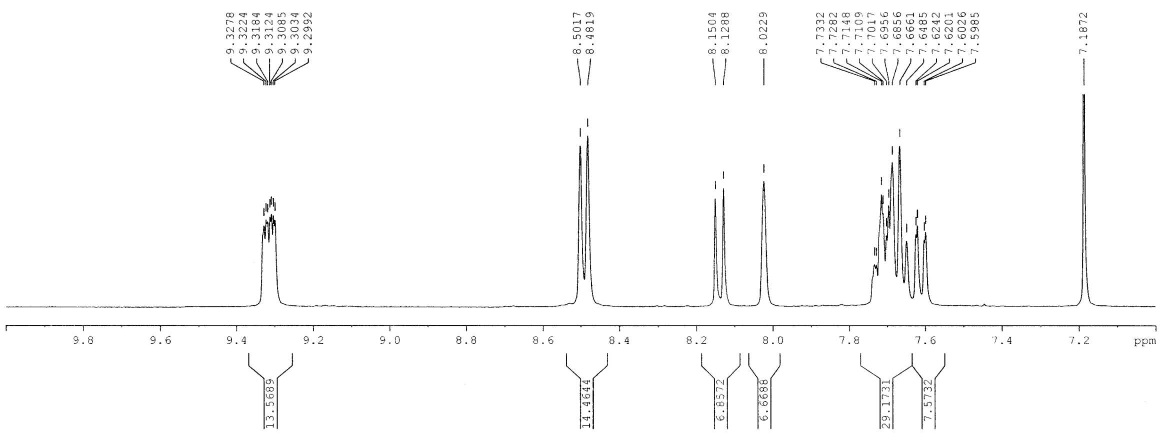

^{13}C spectrum of **DBPh2**

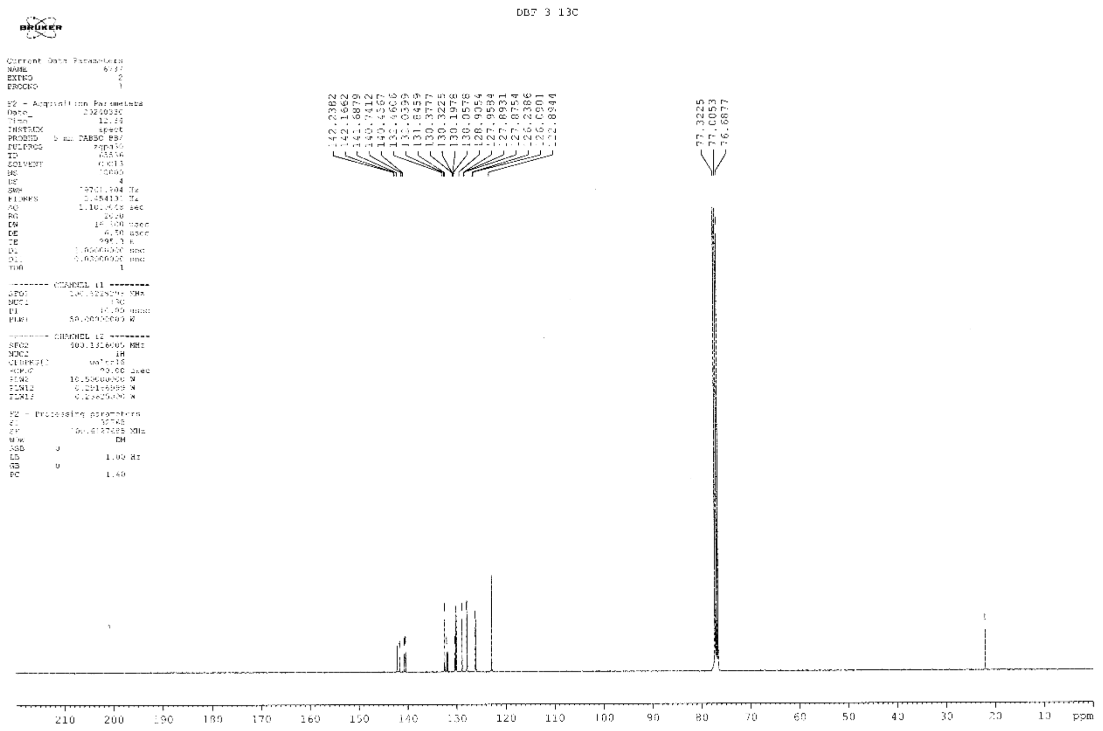

Enlarged spectrum in the range of 120-145 ppm.

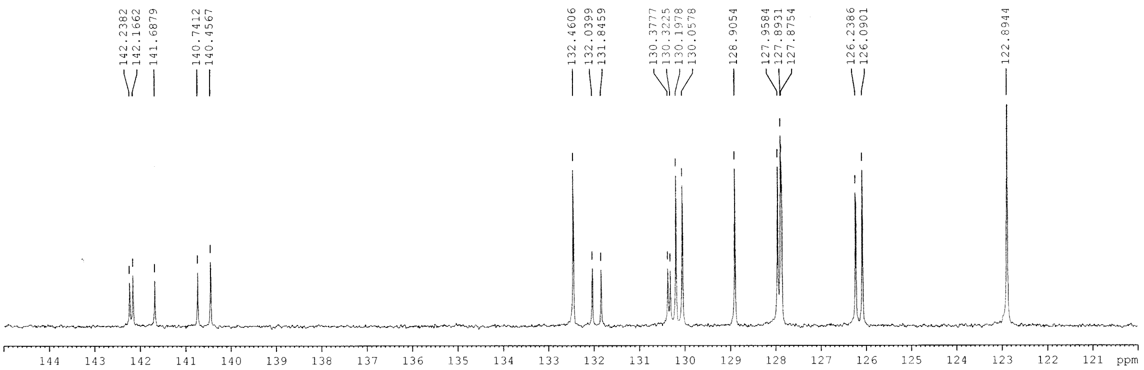

^{1}H spectrum of **DBPh3**

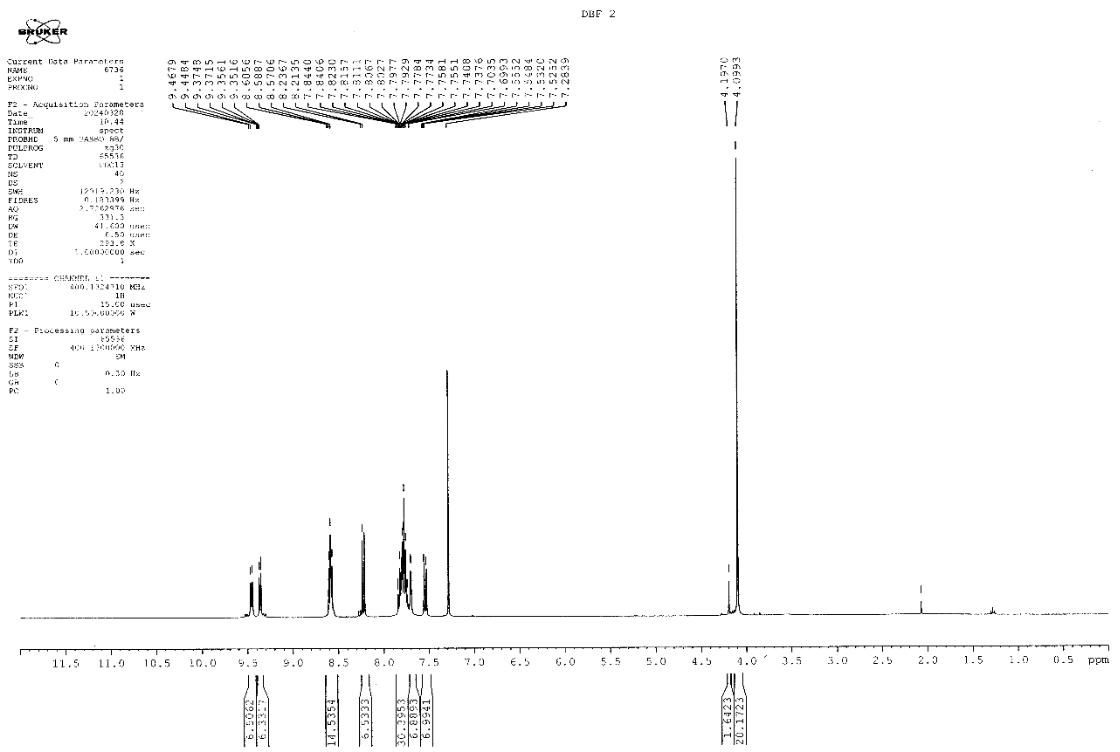

Enlarged spectrum in the range of 7-10 ppm.

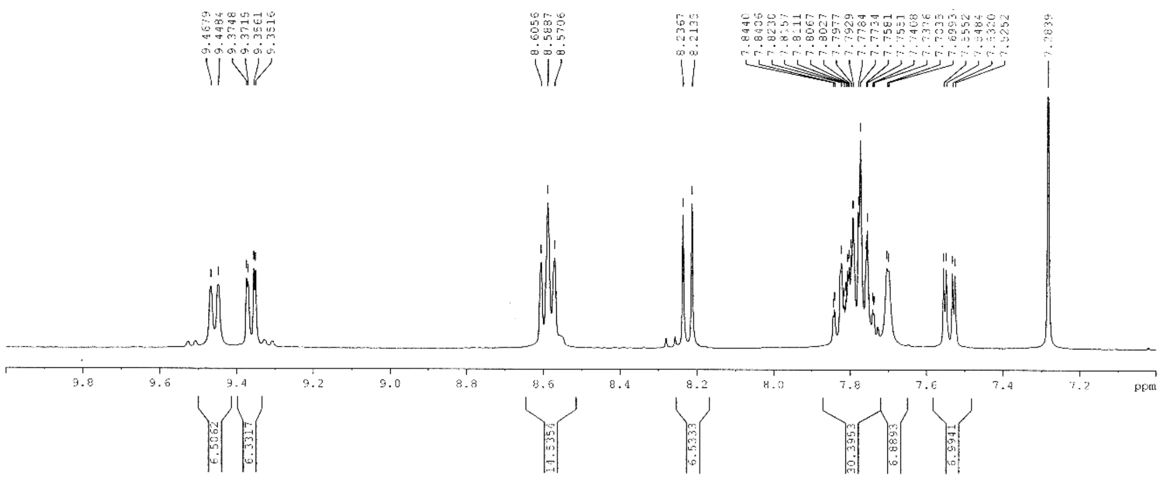

^{13}C spectrum of **DBPh3**

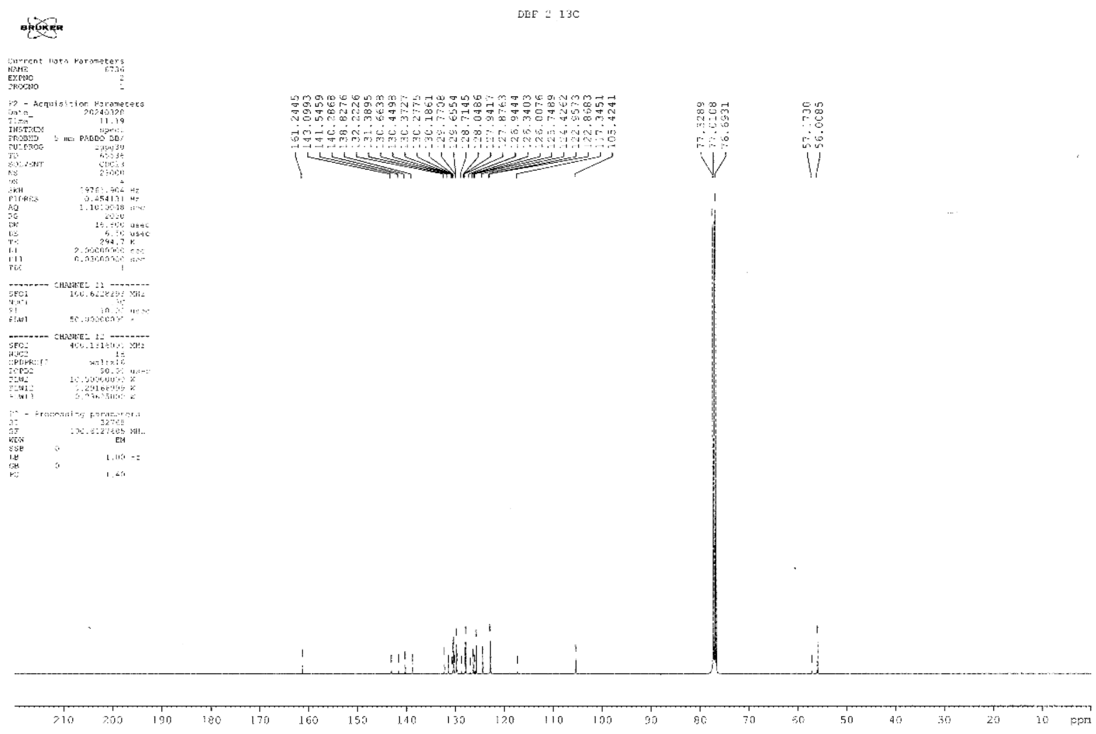

Enlarged spectrum in the range of 50-165 ppm.

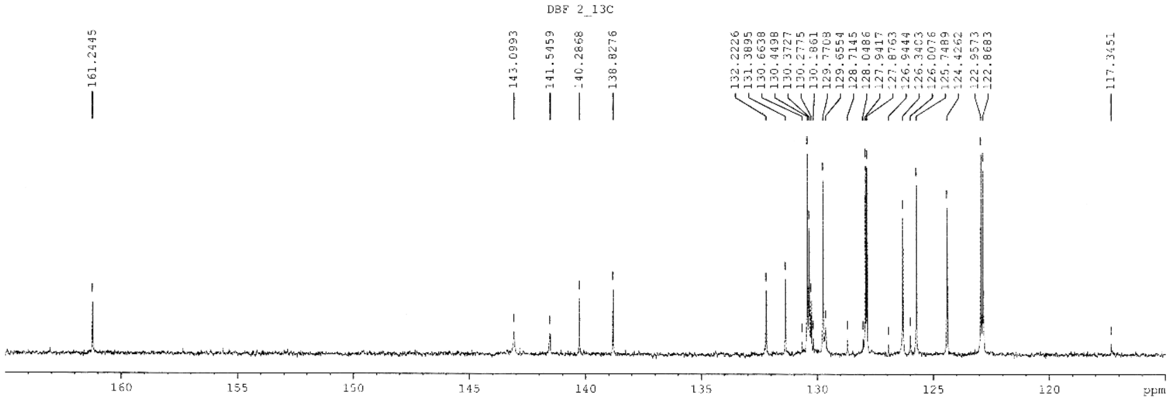

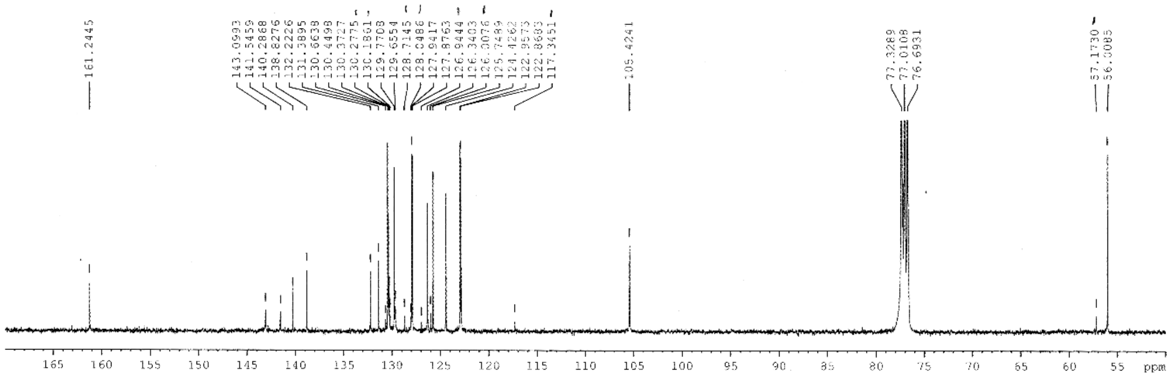

^{1}H spectrum of **DBPh4**

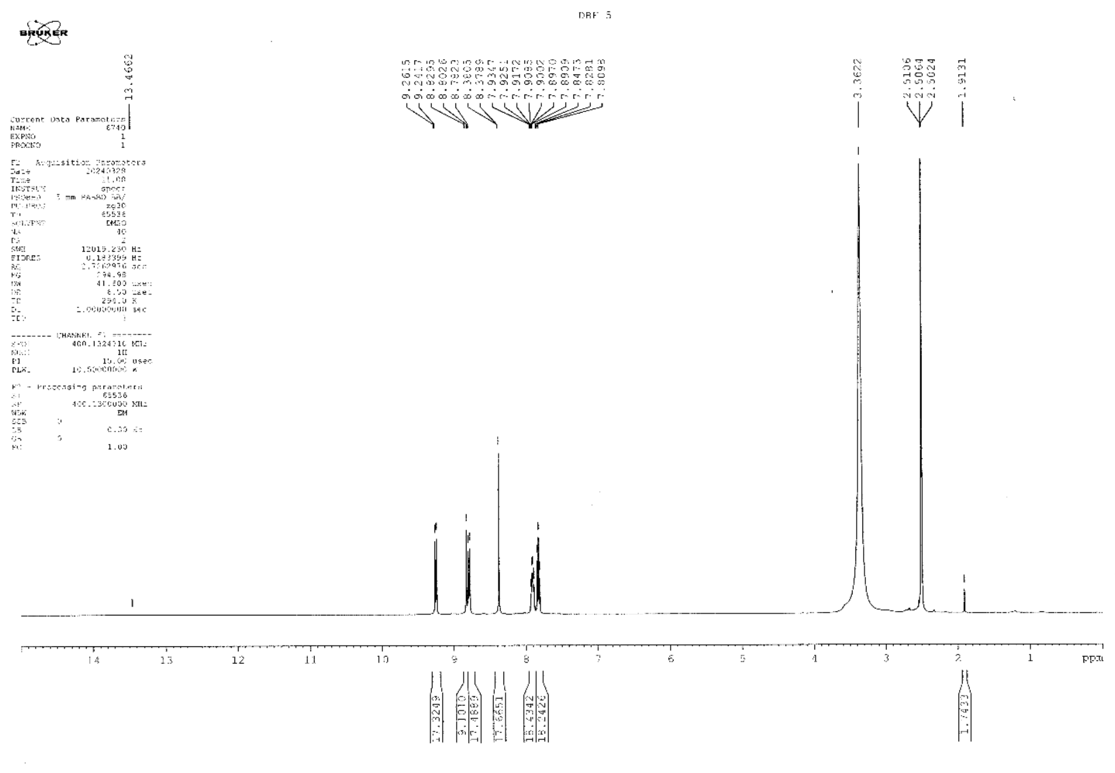

Enlarged spectrum in the range of 7-10 ppm.

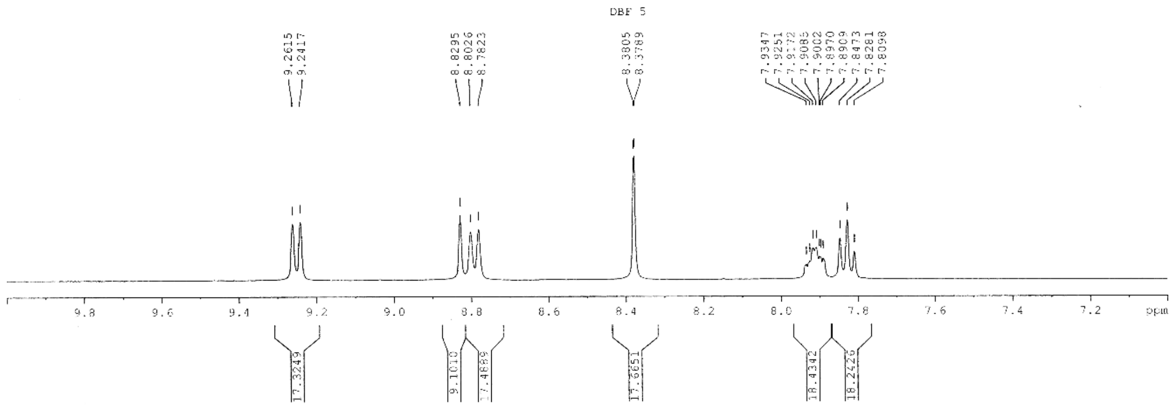

^{13}C spectrum of **DBPh4**

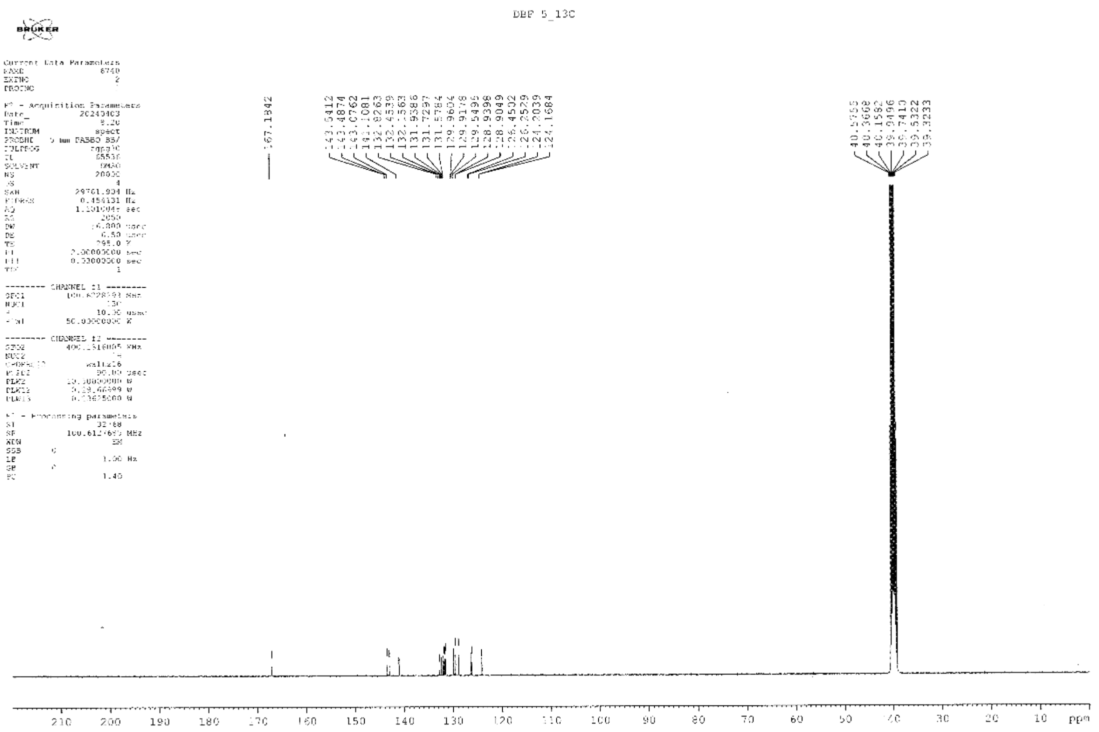

Enlarged spectrum in the range of 120-170 ppm.

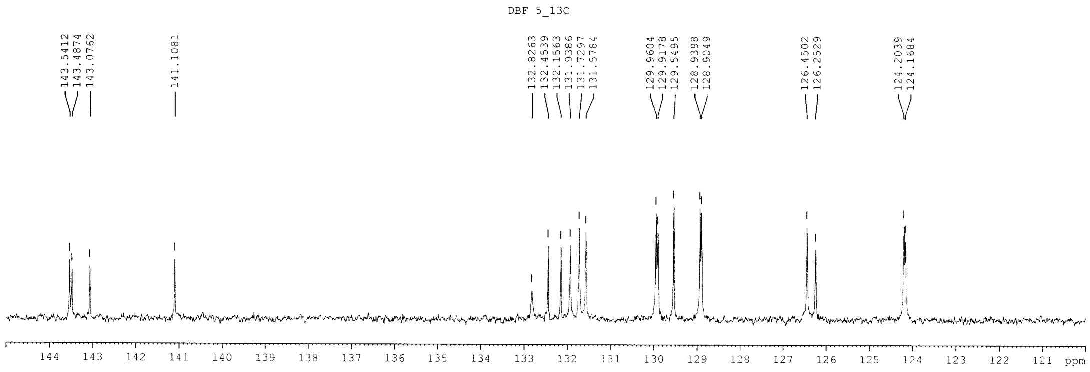

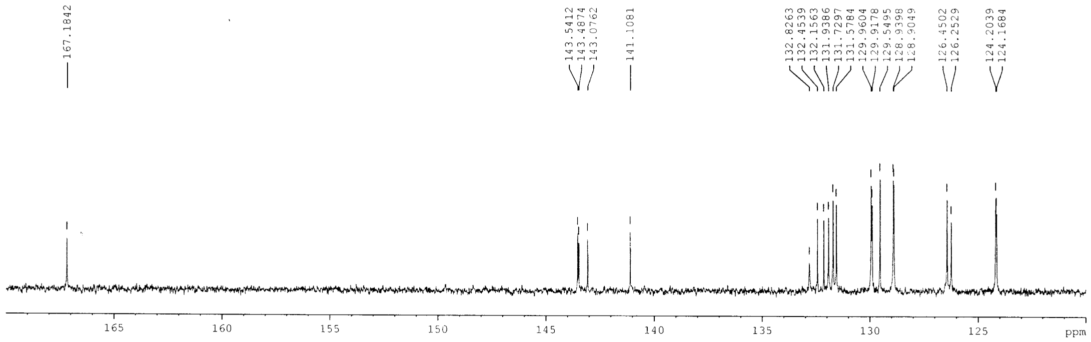

^{1}H spectrum of **DBPh5**

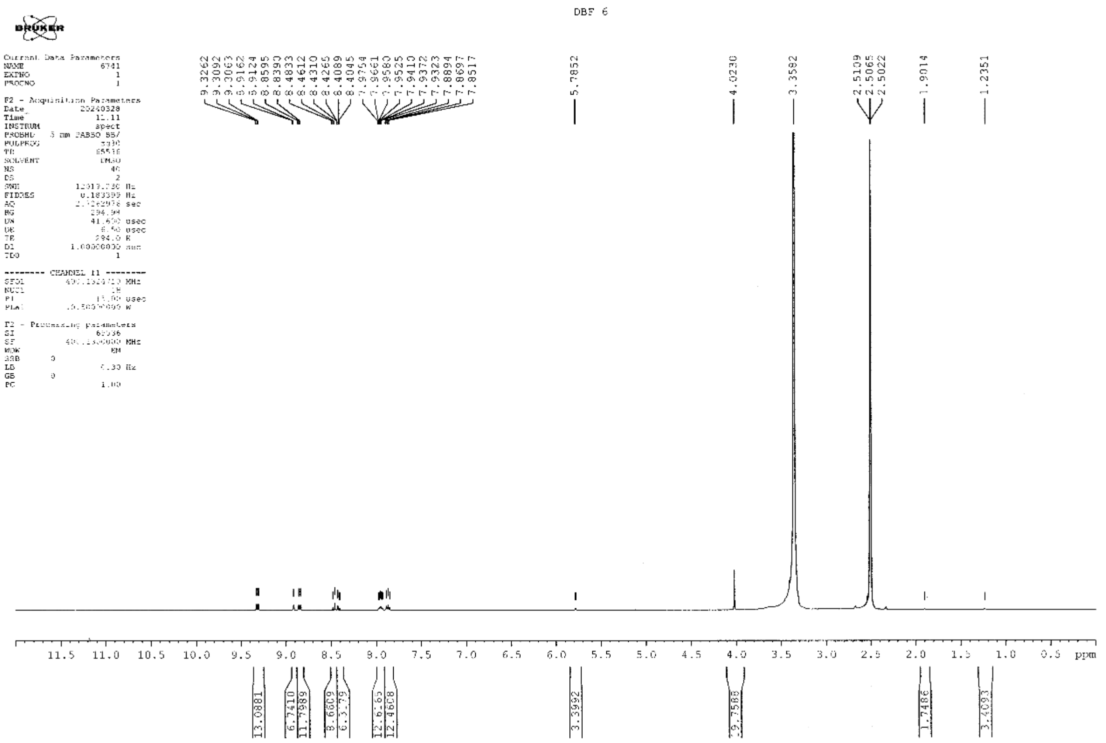

Enlarged spectrum in the range of 7-10 ppm.

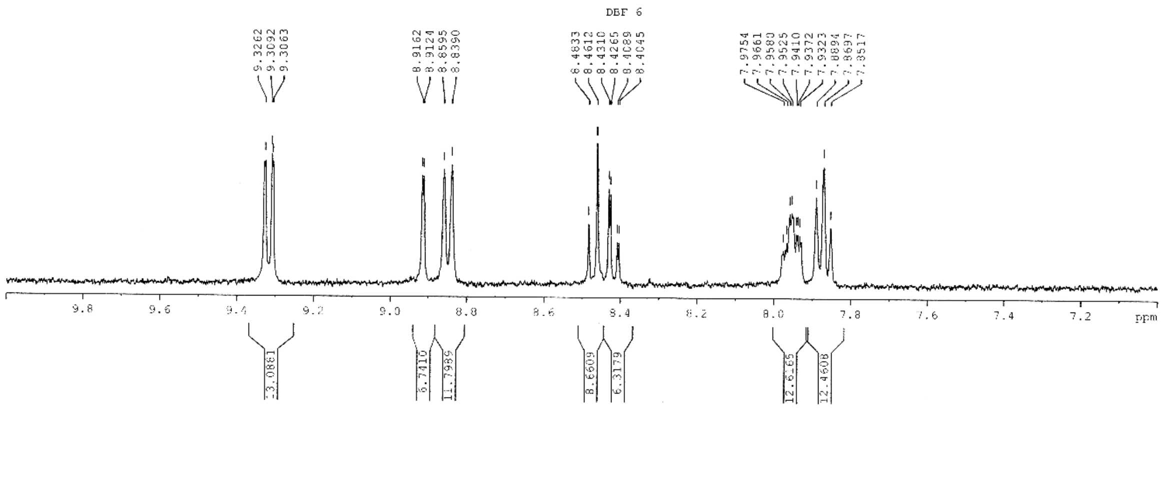

^{1}H spectrum of **DBPh6**

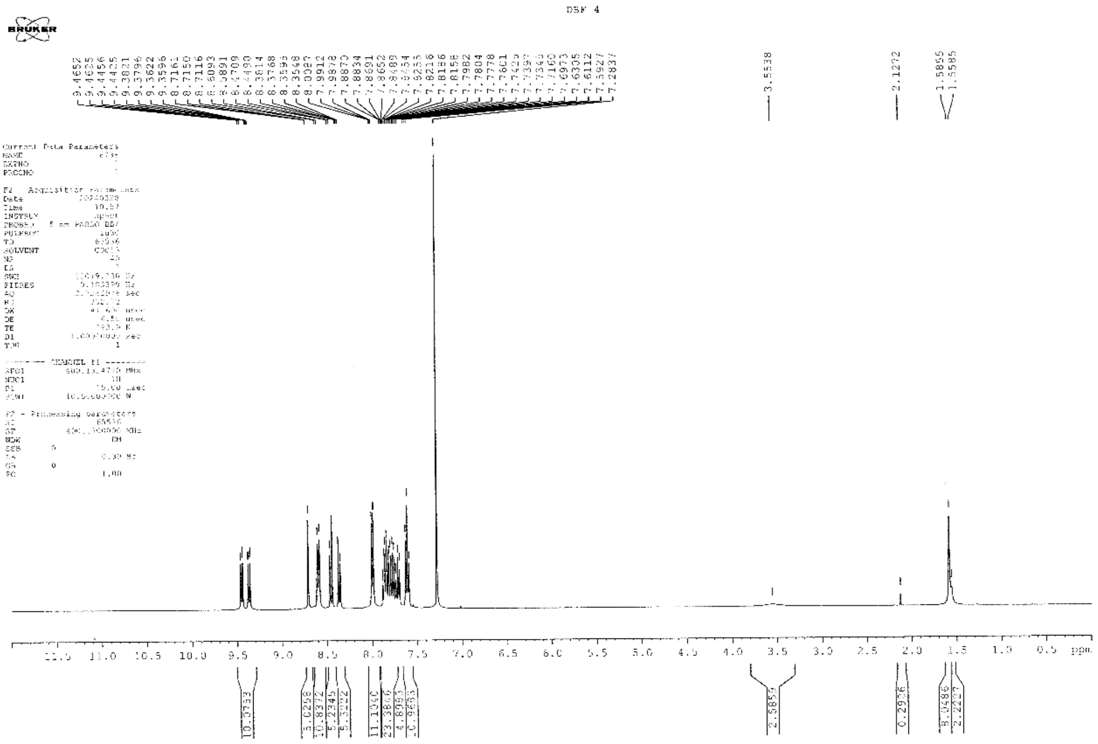

Enlarged spectrum in the range of 7-10 ppm.

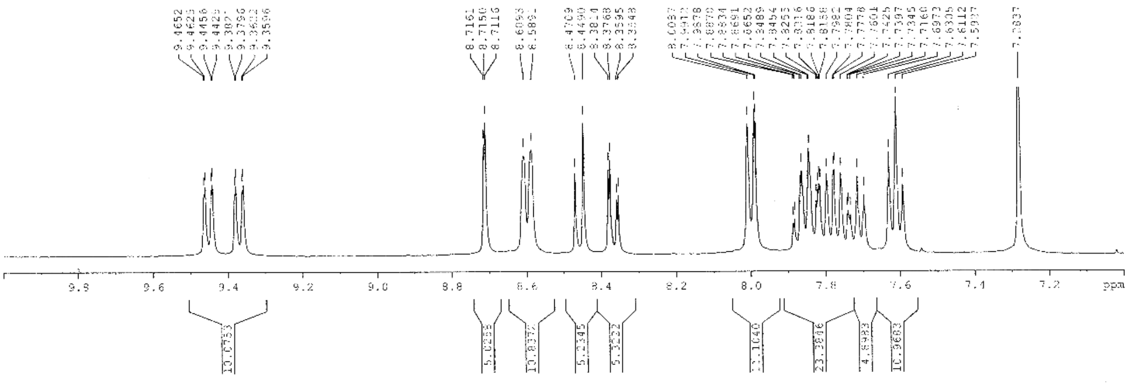

^{13}C spectrum of **DBPh6**

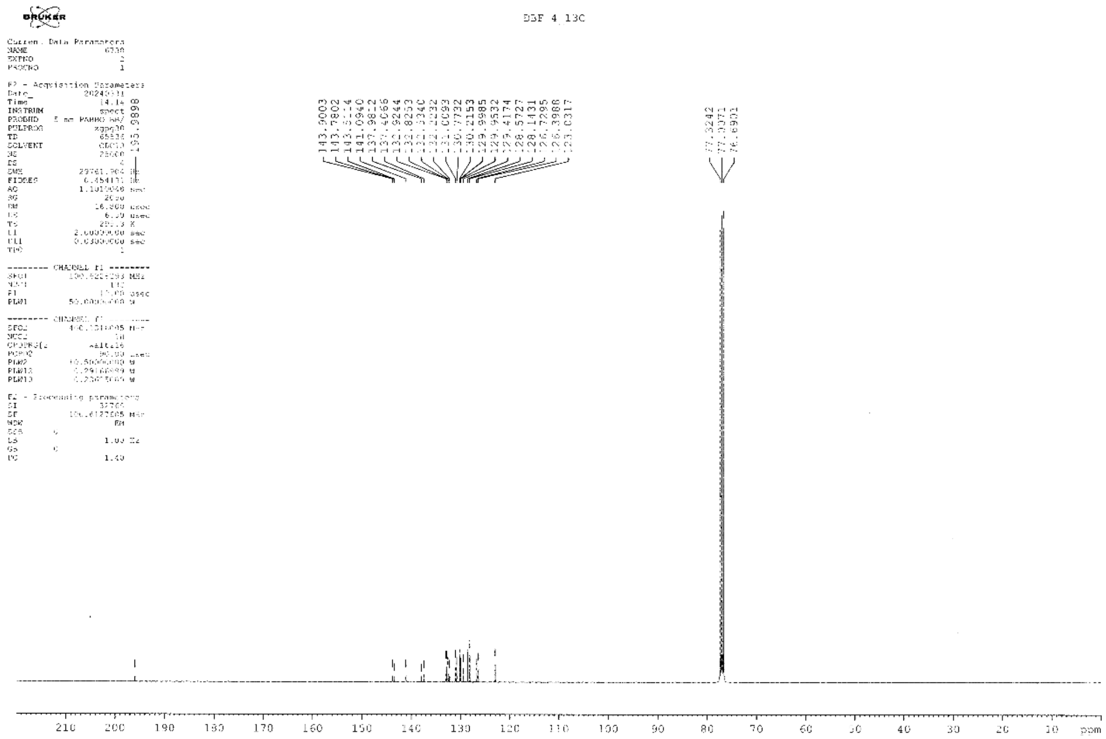

Enlarged spectrum in the range of 100-200 ppm.

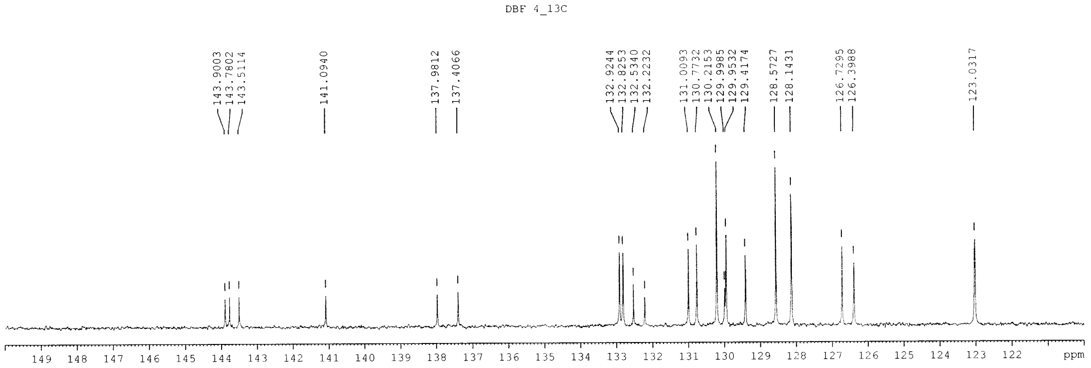

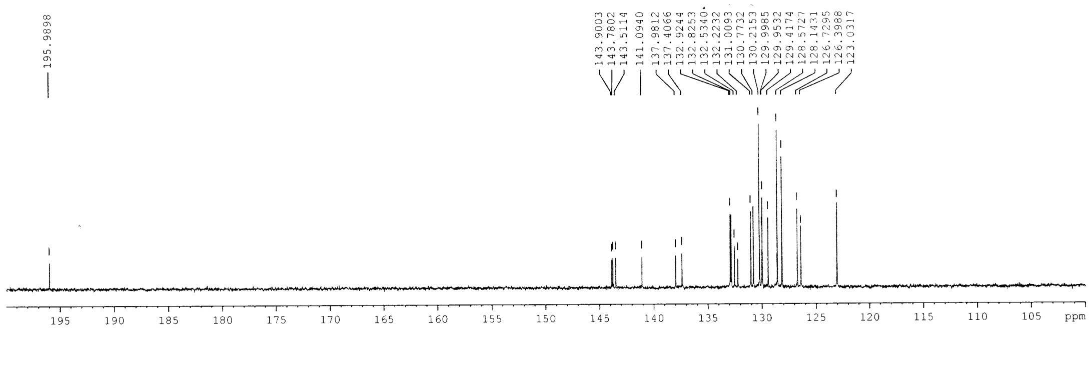

^{1}H spectrum of **DBPh7**

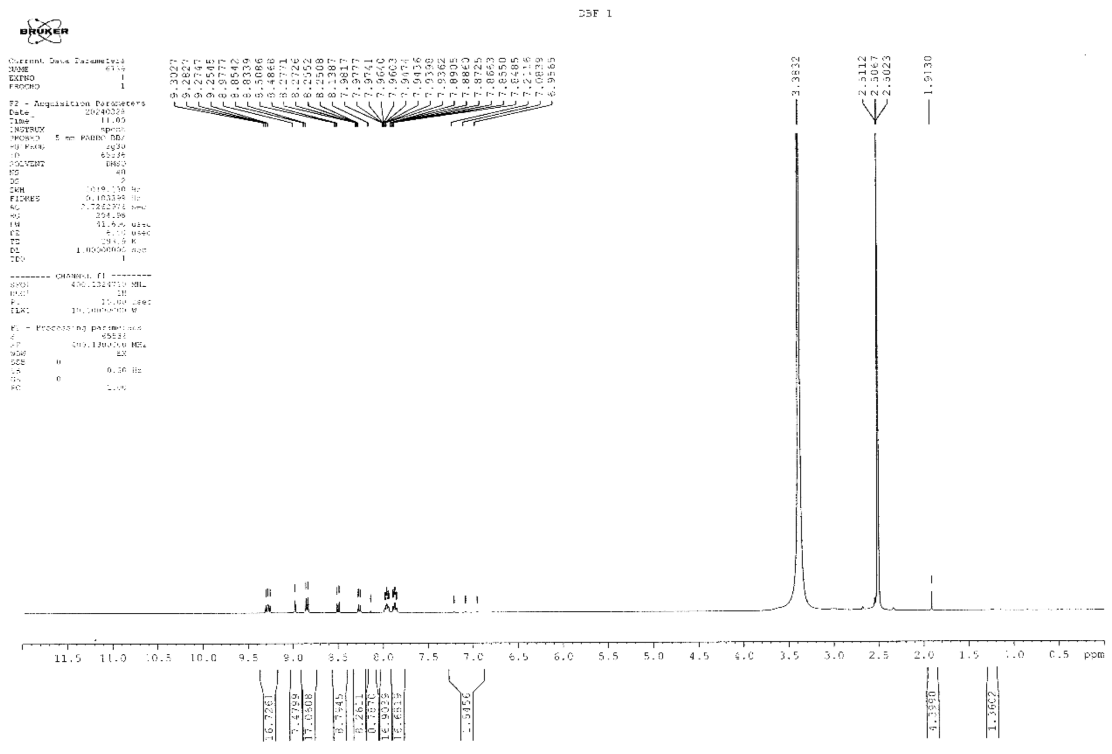

Enlarged spectrum in the range of 7-10 ppm.

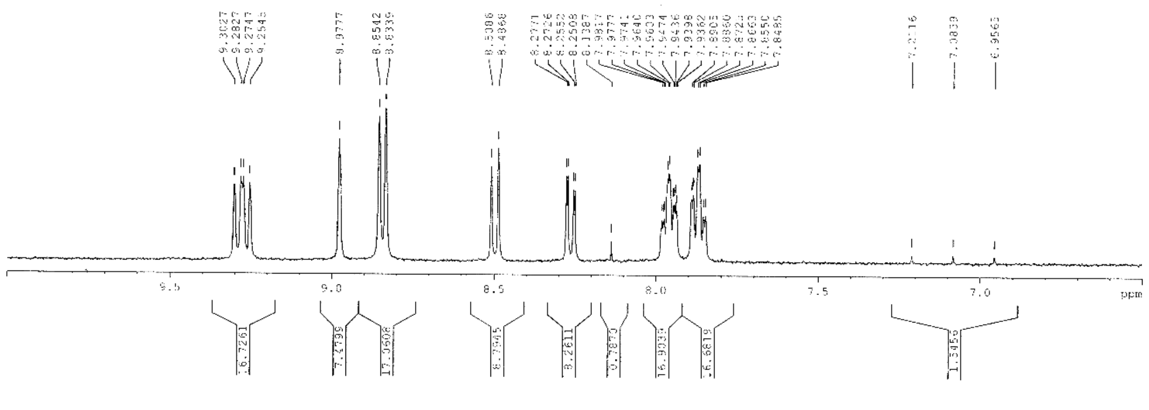

^{13}C spectrum of **DBPh7**

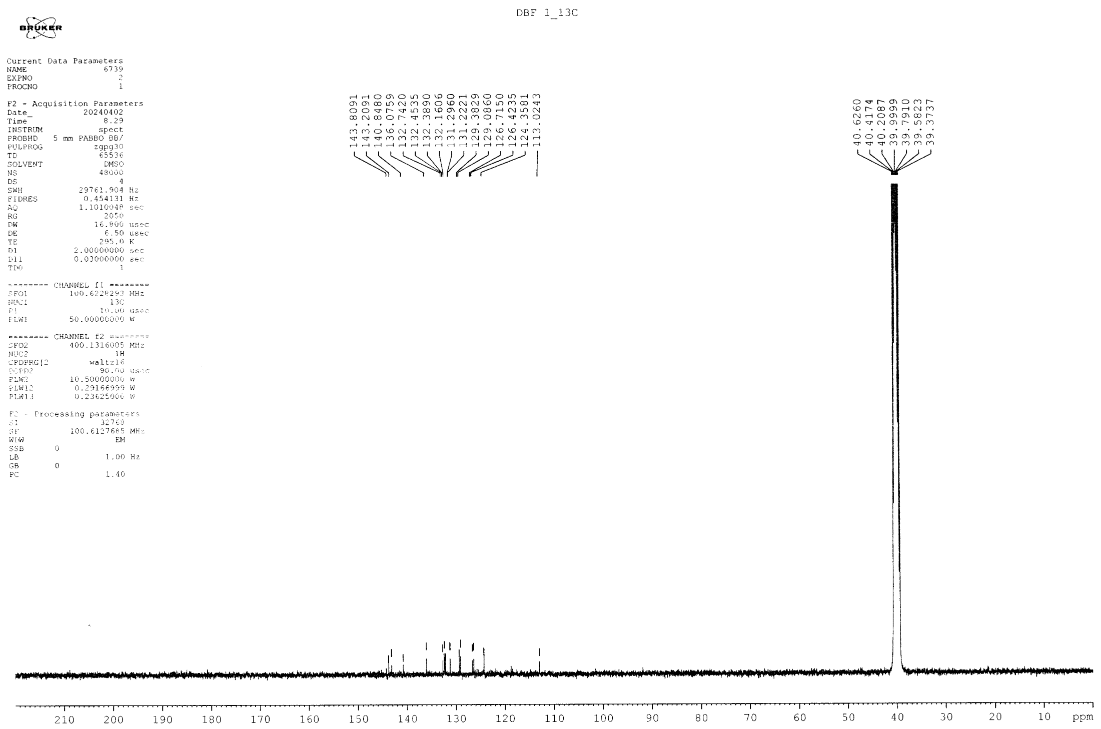

Enlarged spectrum in the range of 100-170 ppm.

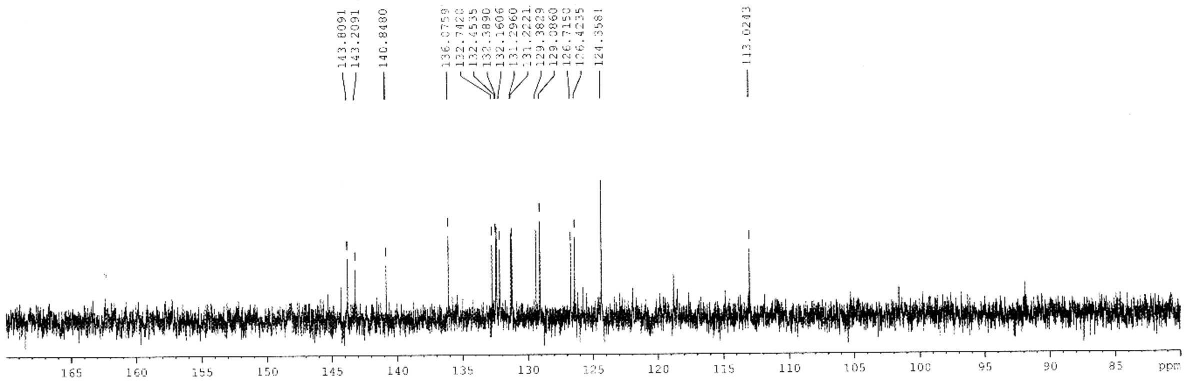

^{1}H spectrum of **DBPh8**

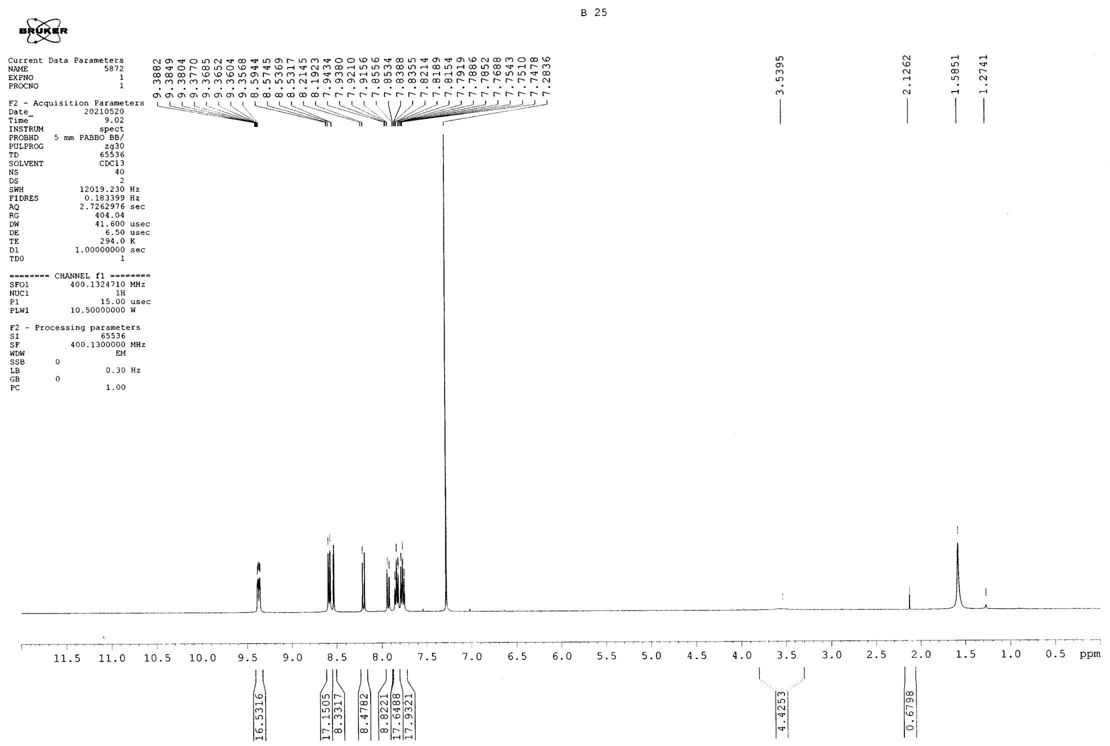

Enlarged spectrum in the range of 6-9 ppm.

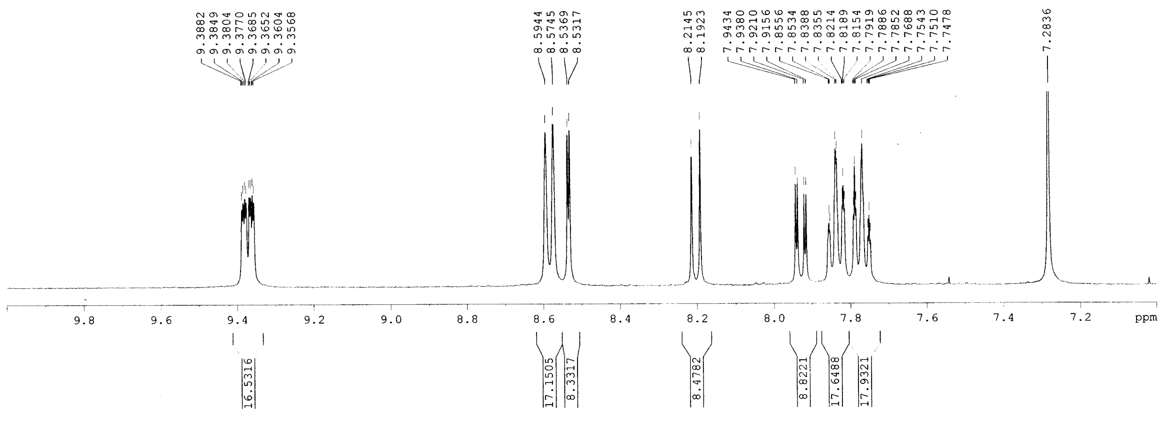

^{13}C spectrum of **DBPh8**

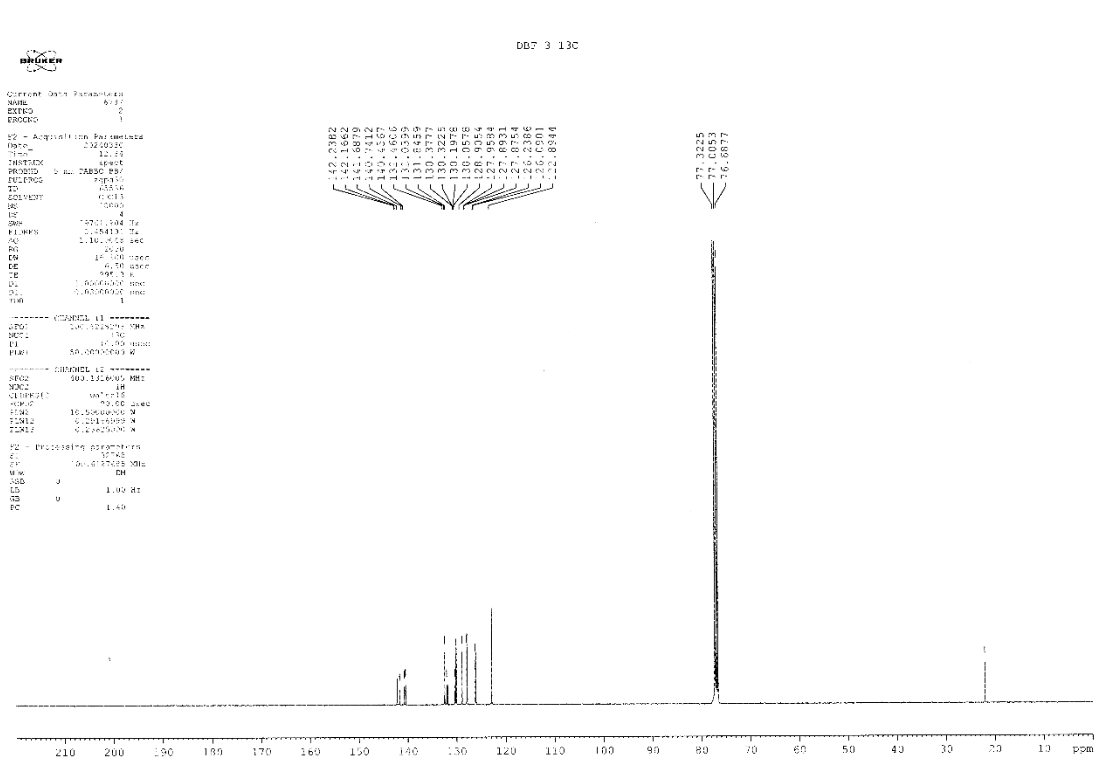

Enlarged spectrum in the range of 110-150 ppm.

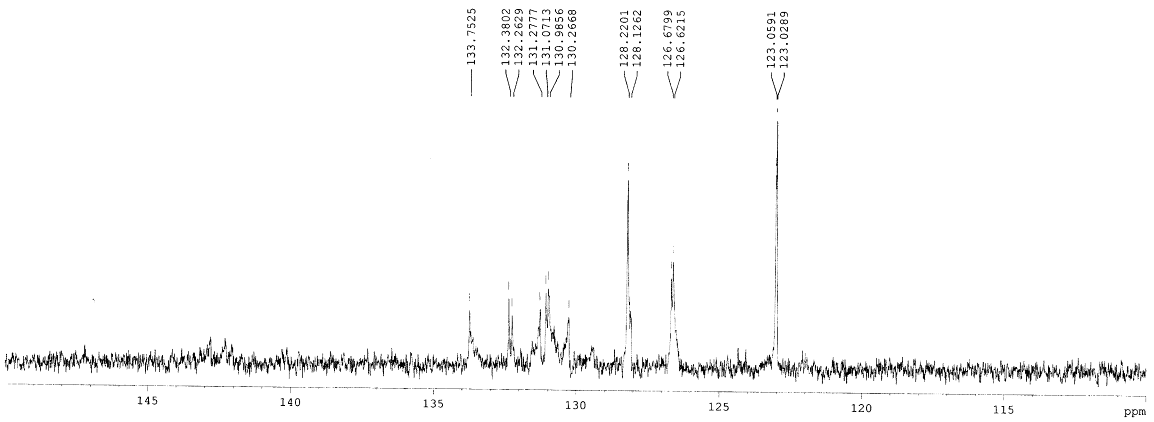

^{1}H spectrum of **DBPh9**

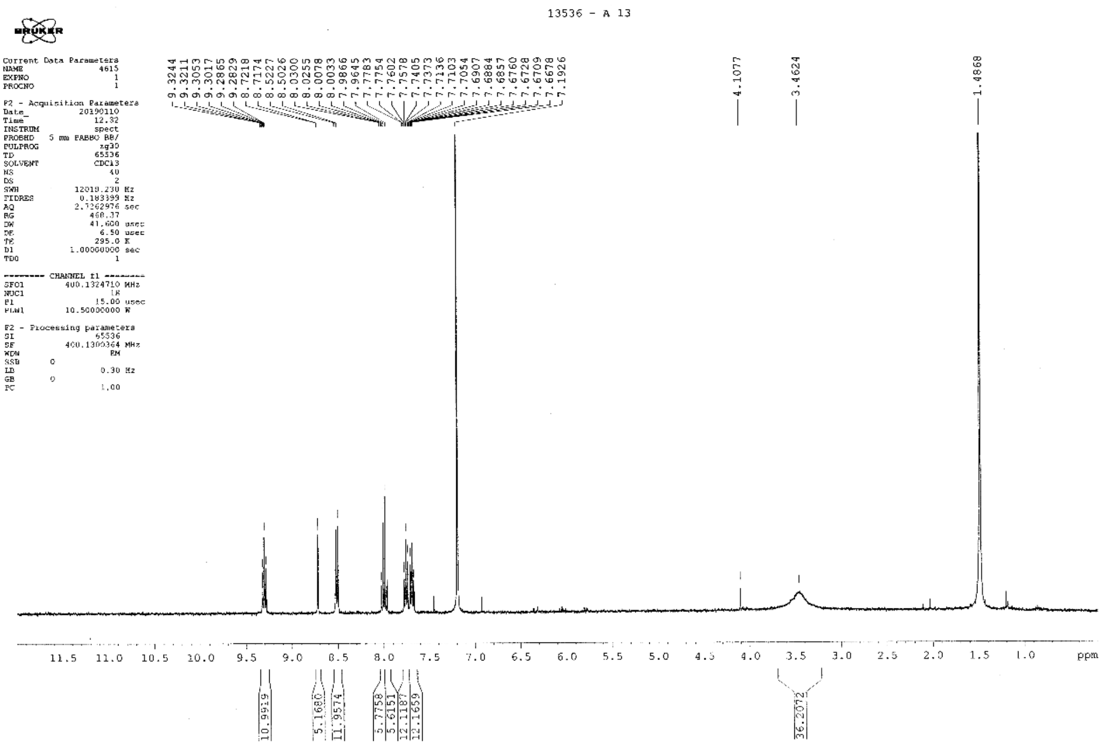

Enlarged spectrum in the range of 7-10ppm.

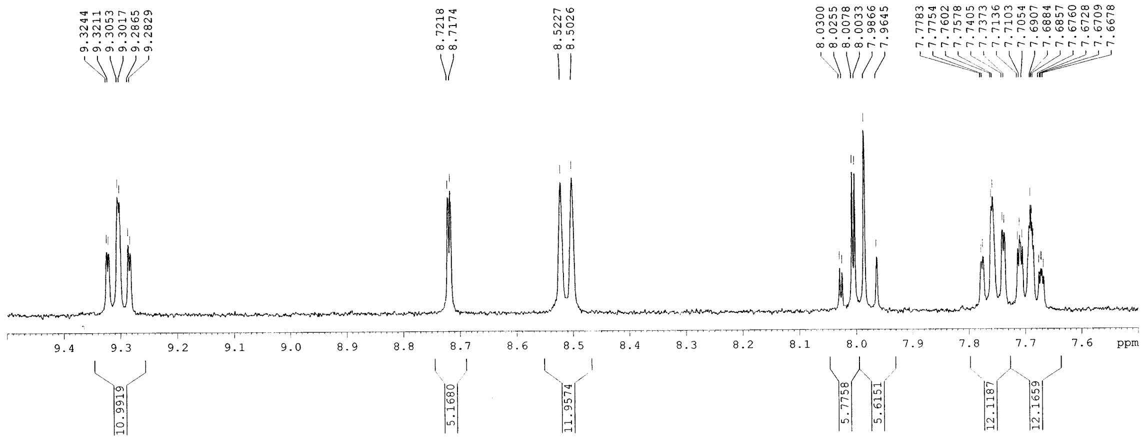

^{13}C spectrum of **DBPh9**

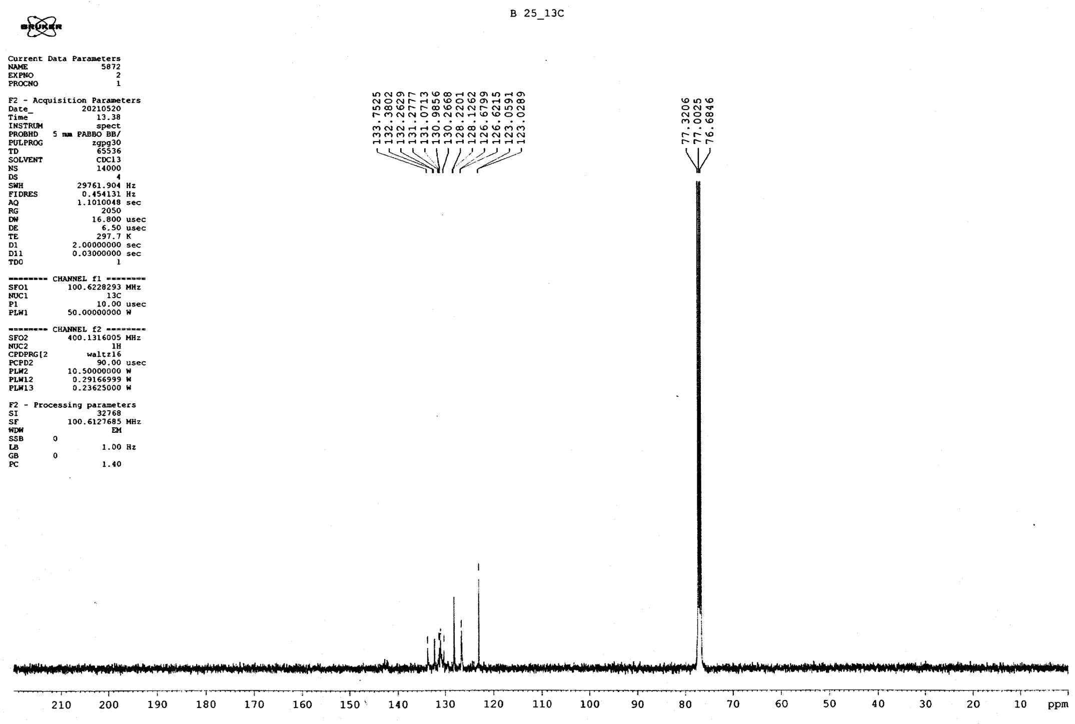

^{1}H NMR spectra of samples containing standards of products formed from (phenylthio)acetic acid (PhTAA) under photoreduction conditions

^{1}H NMR spectrum of (phenylthio)acetic acid (PhTAA)

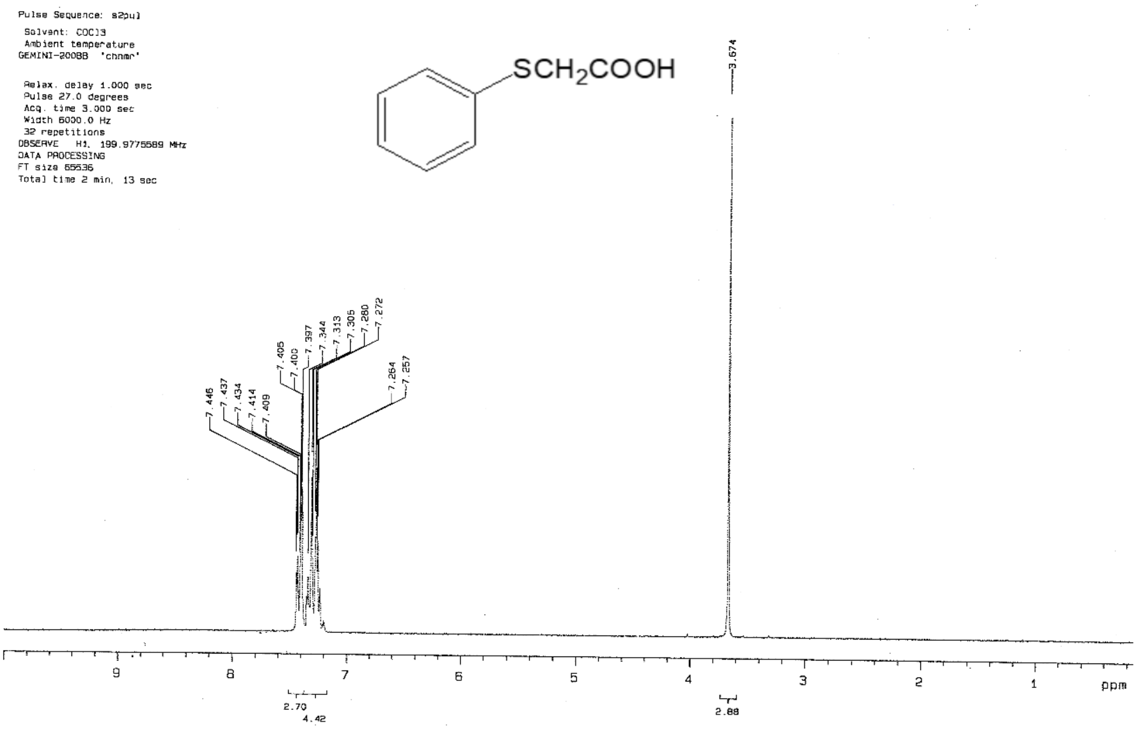

^{1}H NMR spectrum of diphenyl-disulfide

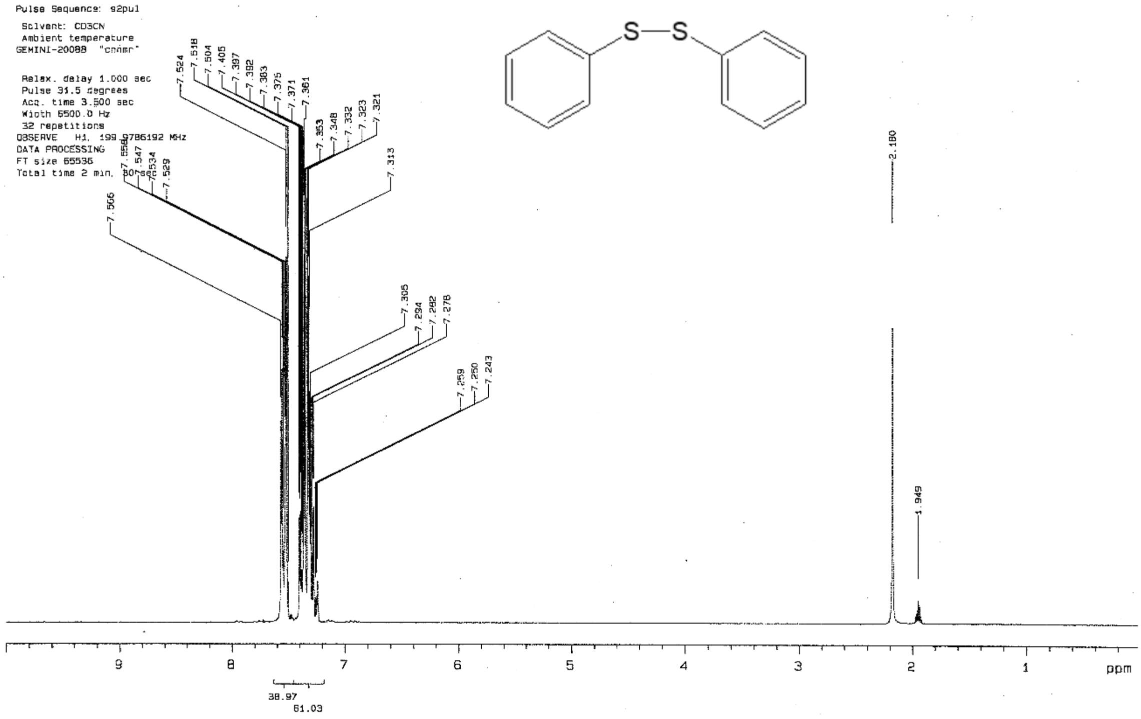

^{1}H NMR spectrum of a sample containing: diphenyl-disulfide, bis(phenylothio)methane

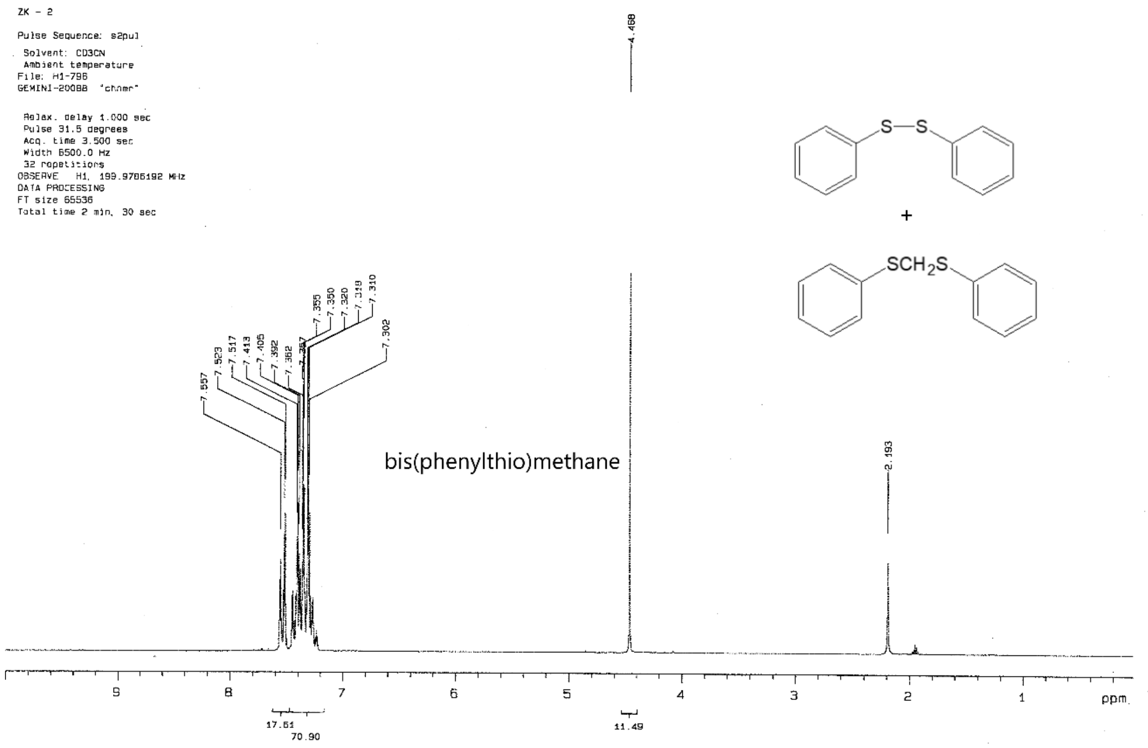

^{1}H NMR spectrum of a sample containing: diphenyl-disulfide, bis(phenylothio)methane, bis(phenylothio)ethane

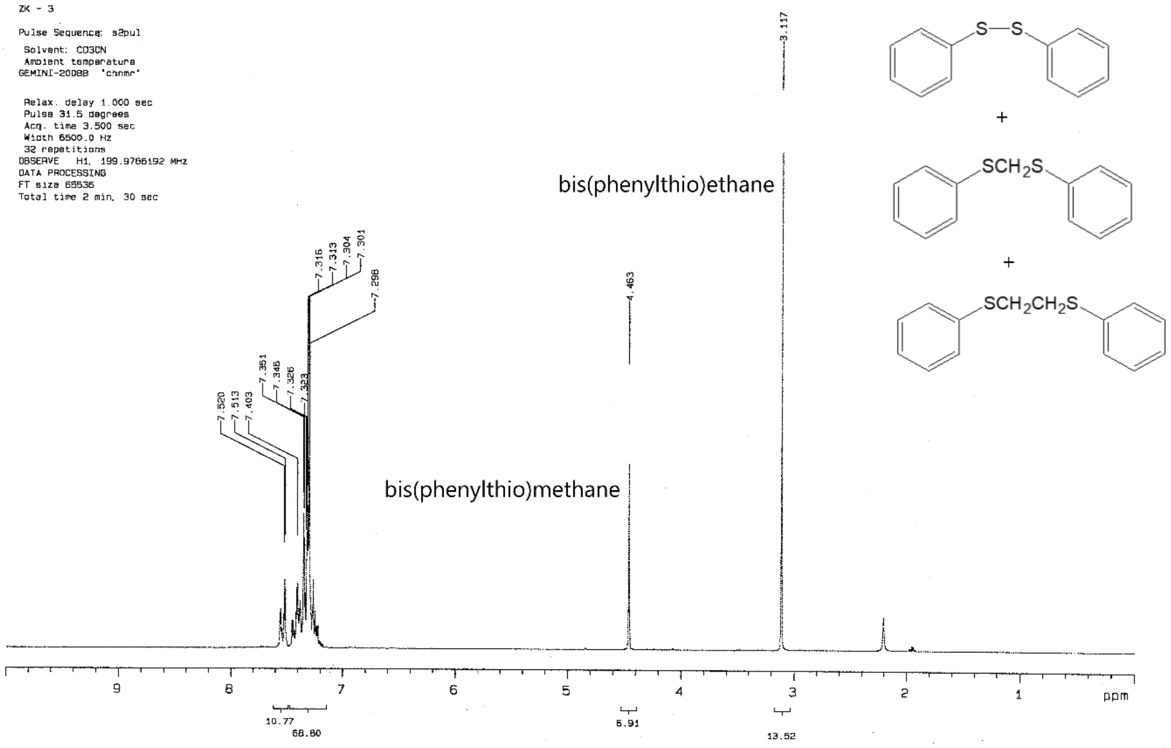

^{1}H NMR spectrum of a sample containing: diphenyl-disulfide, bis(phenylthio)methane, bis(phenylothio)ethane, thiophenol

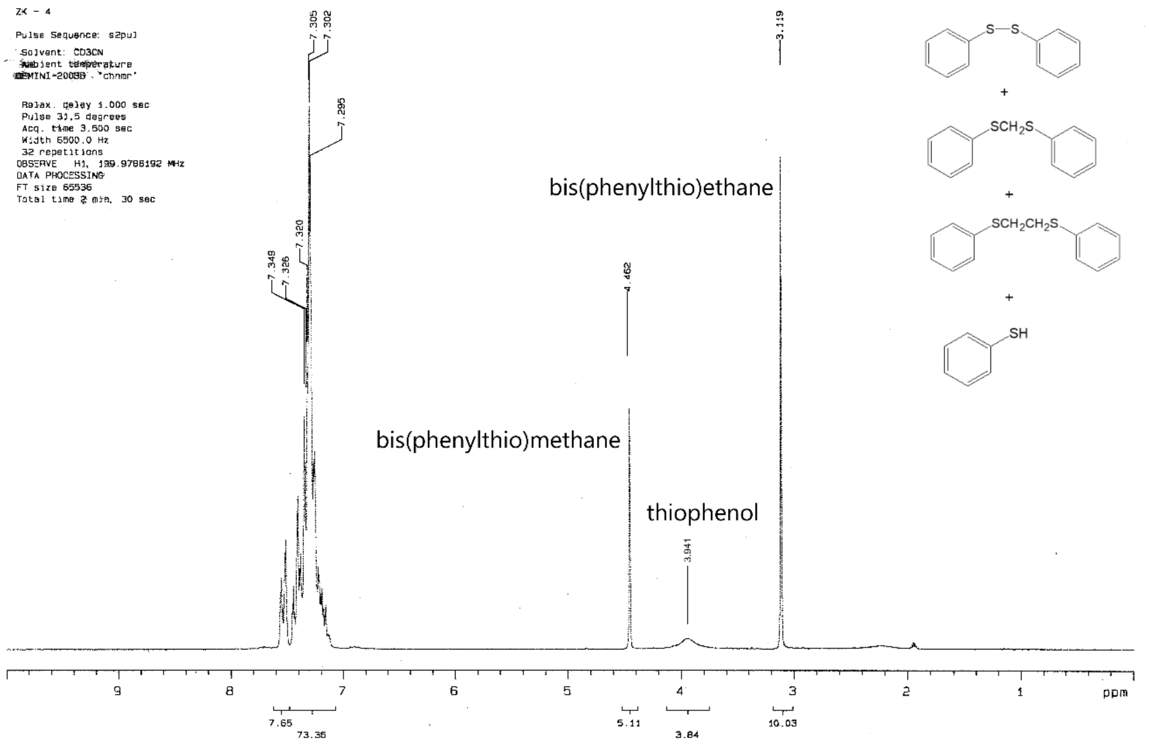

^{1}H NMR spectrum of a sample containing: diphenyl-disulfide, bis(phenylothio)methane, bis(phenylothio)ethane, thiophenol, thioanisole

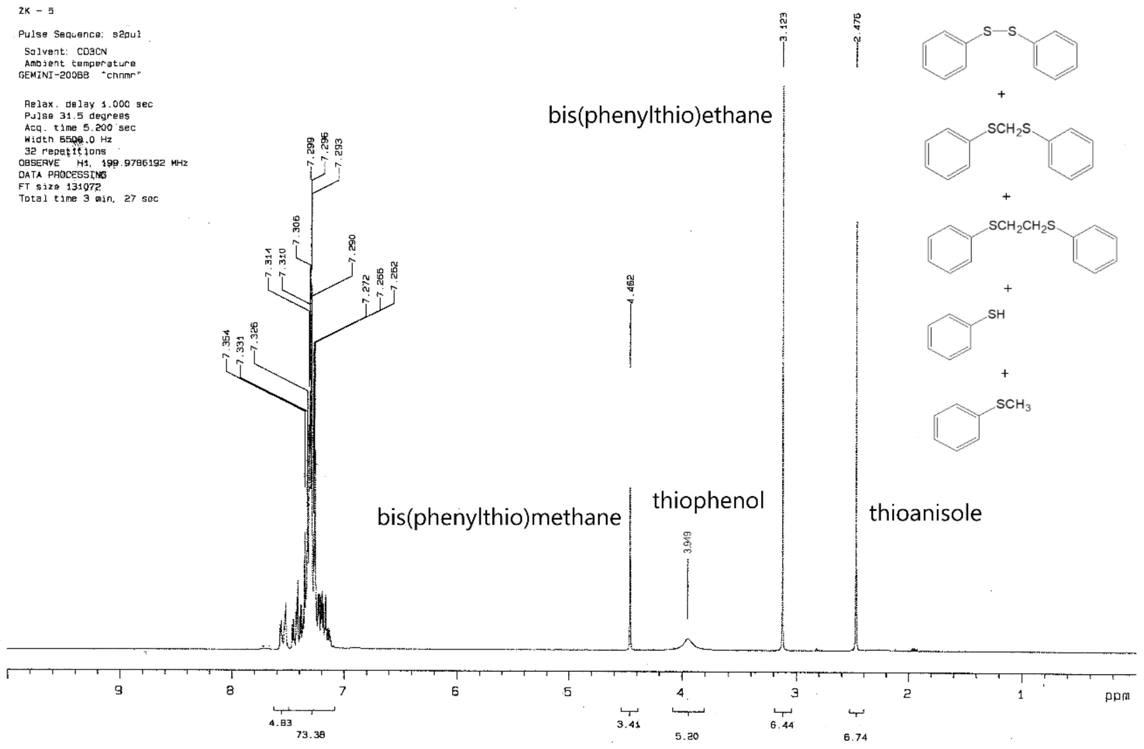

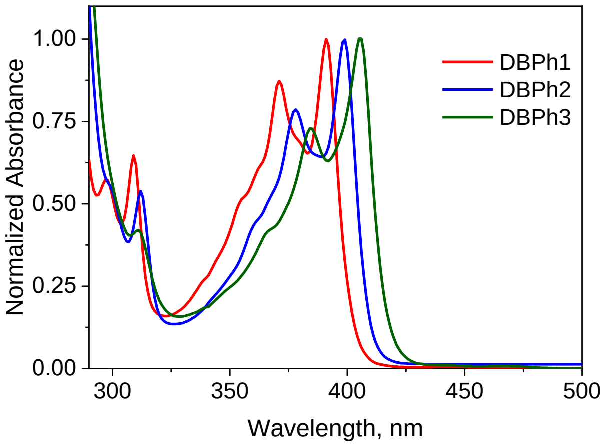

Figure S1. Normalized electronic absorption spectra of the dyes differing in the electron-releasing substituent (DBPh1 vs. DBPH 2 and DBPh3) in ethyl acetate.

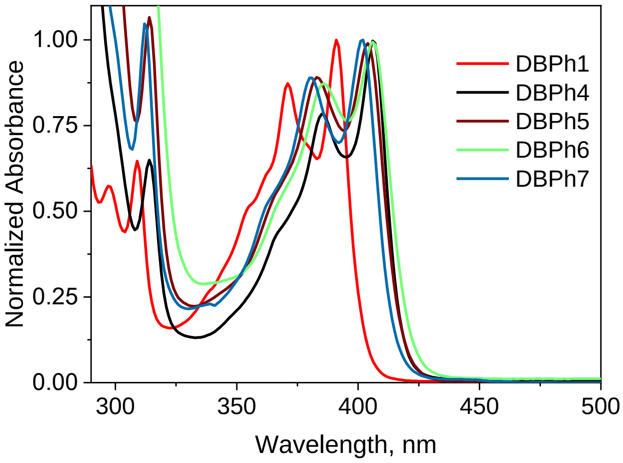

Figure S2. Normalized electronic absorption spectra of dyes differing in the electron-withdrawing substituent (DBPh1 vs. DBPh4–DBPh7) in ethyl acetate.

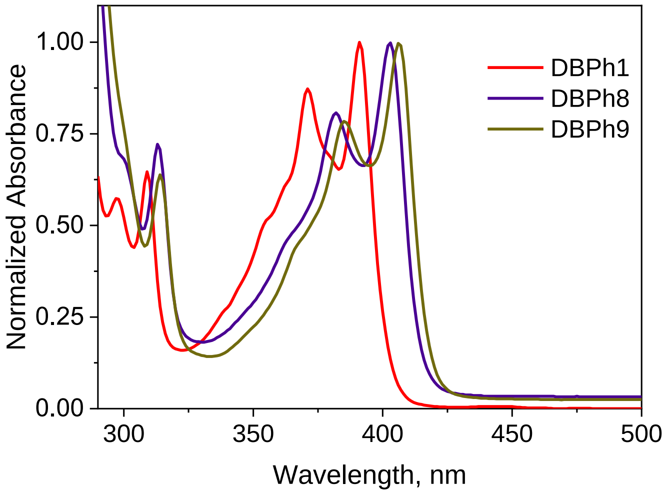

Figure S3. Normalized electronic absorption spectra of dyes differing in heavy atoms (DBPh1 vs. DBPh8 and DBPh9) in ethyl acetate.

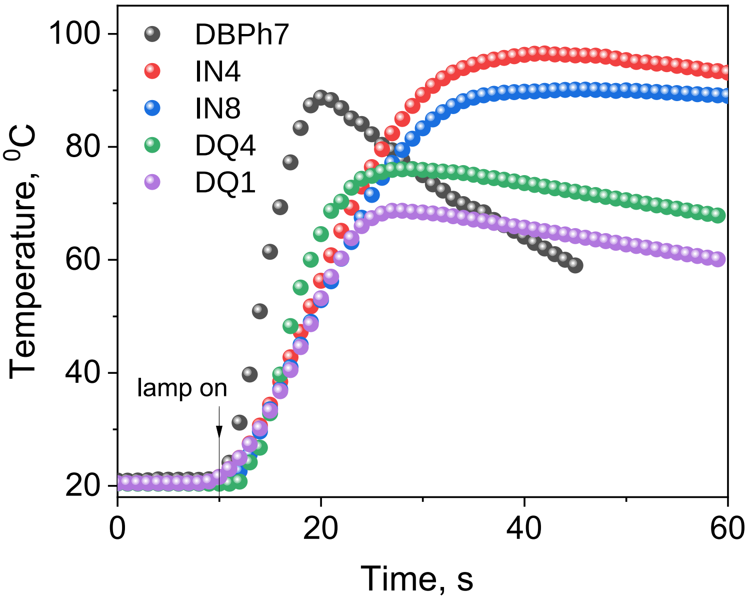

Figure S4. Comparison of kinetic curves of TMPTA polymerization photoinitiated by the tested dibenzo[a,c]phenazine (DBPh7) and other dyes coupled with co-initiator – thiophenoxyacetic acid (0.1 M). The light intensity emitted by the dental lamp was 20 mW cm^{-2}.

Table S1. Initial rate of photoinitiated free radical polymerization of TMPTA (R_p) initiated by different photoredox pairs in the same experimental conditions.

No	Photoinitiators tested	R_p (µmol/s)
DBPh7	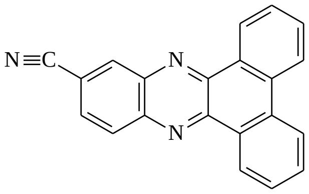 11-Carbonitriledibenzo[a,c]phenazine	194.55
IN4	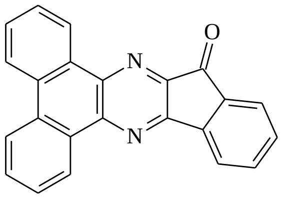 13*H*-Dibenzo[f,h]indeno[1,2-b]quinoxalin-13-one	104.30
IN8	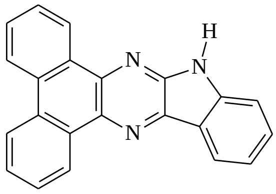 10*H*-Dibenzo[f,h]indolo[2,3-b]quinoxaline	88.50

DQ1	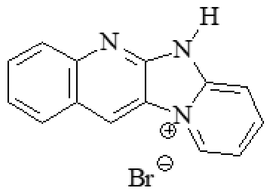<br>Quinoline[2,3-b]-1*H*-imidazo[1,2-a]pyridinium bromide	109.06
DQ4	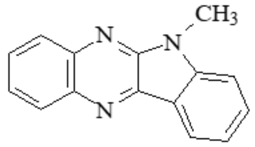<br>6-Methyl-6*H*-indolo[2,3-b]quinoxaline	115.30

References:

a) Pyszka, I.; Jędrzejewska, B. Design of Dyes Based on the Quinoline or Quinoxaline Skeleton towards Visible Light Photoinitiators. *International Journal of Molecular Sciences* **2024**, *25*, doi:10.3390/ijms25084289.

b) Kucybała, Z.; Pyszka, I.; Pączkowski, J. Development of New Dyeing Photoinitiators for Free Radical Polymerization Based on the 1H-Pyrazolo[3,4-b]Quinoxaline Skeleton. Part 2. *J. Chem. Soc., Perkin Trans. 2* **2000**, 1559-1567, doi:10.1039/B000583P.

c) Pyszka, I.; Kucybała, Z. The Effect of Co-Initiator Structure on Photoinitiating Efficiency of Photoredox Couples Composed of Quinoline[2,3-b]-1H-Imidazo[1,2-a]Pyridiniumbromide and Phenoxyacetic Acid or *N,N*-Dimethylaniline Derivatives. *Polymer Bulletin* **2008**, *61*, 553–562, doi:10.1007/s00289-008-0978-7.

d) Pyszka, I.; Kucybała, Z. Quinolineimidazopyridinium Derivatives as Visible-Light Photoinitiators of Free Radical Polymerization. *Polymer* **2007**, *48*, 959–965, doi:10.1016/j.polymer.2006.07.014.

e) Pyszka, I.; Skowroński, Ł.; Jędrzejewska, B. Study on New Dental Materials Containing Quinoxaline-Based Photoinitiators in Terms of Exothermicity of the Photopolymerization Process. *International Journal of Molecular Sciences* **2023**, *24*, doi:10.3390/ijms24032752.

f) Pyszka I, Jędrzejewska B. Acenaphthoquinoxaline Derivatives as Dental Photoinitiators of Acrylates Polymerization. Materials **2021,** *14*. https://doi.org/10.3390/ma14174881.

g) Pyszka I, Jędrzejewska B, Kucybała Z. Effective Singlet Oxygen Sensitizers Based on the Phenazine Skeleton as Efficient Light Absorbers in Dye Photoinitiating Systems for Radical Polymerization of Acrylates, *Materials* **2021**, *14*, 3085, doi:10.3390/ma14113085
